# Molecular Interactions Governing the Rat Aryl Hydrocarbon Receptor Activities of Polycyclic Aromatic Compounds and Predictive Model Development

**DOI:** 10.3390/molecules29194619

**Published:** 2024-09-29

**Authors:** Lingmin Jin, Bangyu Chen, Guangcai Ma, Xiaoxuan Wei, Haiying Yu

**Affiliations:** 1 College of Geography and Environmental Sciences, Zhejiang Normal University, Jinhua 321004, China; jlm3630@163.com (L.J.); chenbangyuu@163.com (B.C.); magc@zjnu.edu.cn (G.M.); xxwei@zjnu.edu.cn (X.W.); 2 School of Environment Science and Spatial Informatics, China University of Mining and Technology, Xuzhou 221116, China

**Keywords:** polycyclic aromatic compounds, rat aryl hydrocarbon receptor activity, molecular docking, molecular dynamics simulations, QSAR model

## Abstract

Polycyclic aromatic compounds (PACs) exhibit rat aryl hydrocarbon receptor (rAhR) activities, leading to diverse biological or toxic effects. In this study, the key amino residues and molecular interactions that govern the rAhR activity of PACs were investigated using in silico strategies. The homology model of rAhR was first docked with 90 PACs to yield complexes, and the results of the molecular dynamics simulations of 16 typical complexes showed that the binding energies of the complexes range from −7.37 to −26.39 kcal/mol. The major contribution to the molecular interaction comes from van der Waals forces, and Pro295 and Arg316 become the key residues involved in most complexes. Two QSAR models were further developed to predict the rAhR activity of PACs (in terms of log IEQ for PACs without halogen substitutions and log%-TCDD-max for halogenated PACs). Both models have good predictive ability, robustness, and extrapolation ability. Molecular polarizability, electronegativity, size, and nucleophilicity are identified as the important factors affecting the rAhR activity of PACs. The developed models could be employed to predict the rAhR activity of other reactive PACs. This work provides insight into the mechanisms and interactions of the rAhR activity of PACs and assists in the assessment of their fate and risk in organisms.

## 1. Introduction

Polycyclic aromatic hydrocarbons (PAHs) are a group of widespread organic compounds in various media [1,2,3]. In recent years, different types of PAH derivatives have been determined to be the accompaniment of the parent PAHs [4,5,6], such as methylated PAHs (M-PAHs), oxygenated PAHs (O-PAHs), nitrated PAHs (N-PAHs), chlorinated PAHs (Cl-PAHs), and brominated PAHs (Br-PAHs), referred to as polycyclic aromatic compounds (PACs). Previous experiments have suggested that exposure to many reactive PACs can enhance oxidative stress and even produce mutagenicity and carcinogenicity [3,4,7,8].

The aryl hydrocarbon receptor (AhR) belongs to the basic helix–loop–helix/Per-Arnt-Sim (bHLH/PAS) protein family [9]. In the PAS domain of AhR, PAS B functions as a ligand-binding domain (LBD), which binds with ligands to form ligand–AhR complexes, resulting in the conformational transition of AhRs [10]. An activated AhR can influence the expression of multiple genes and induce diverse biological or toxic effects [9,11]. It has been proven that many PACs, including M-PAHs, O-PAHs, and N-PAHs, can bind with rat AhR (rAhR) and activate luciferase, causing AhR activity in a rat H4IIE-*luc* bioassay [12,13,14,15,16]. The results showed that M-PAHs were more potent than their parent compounds [13], and the oxidation of M-PAHs seemed to increase the AhR-mediated potency of the compounds, with 2-methylanthracene-9,10-dione being almost twice as potent as 2-methylanthracene [14]. Moreover, O-PAHs (PAH ketones and quinones) have more significant AhR activities than parent PAHs [11,17]. As a rodent who is closely homologous to humans, the rat is an important biological model in toxicology research. Comparisons for AhR genomic sequences [18] and studies summarizing the responses to various reference compounds in rat H4IIe cells and human HepG2 cells [19] have shown that the rat AhR signaling pathway is easier to activate using ligands than human AhRs are. Human AhR-mediated *β*-galactosidase activity for PACs has also been assessed in *lacZ* reporter gene assays using yeast *Saccharomyces cerevisiae* YCM3 cells [11], and PAH ketones and quinones showed significantly stronger activities than benzo[a]pyrene. The comparison of AhR activities among halogenated PAHs indicated that Cl-PAHs yielded stronger AhR activities, resulting in approximately 30–50 times higher toxicity being mediated through AhR activation than through dioxins [20]. The AhR activity of Cl-PAHs tended to increase with the number of chlorine atoms on the corresponding parent PAH skeletons [20]. These bioassay results confirmed the AhR activities of several PACs in both rats and humans. However, the mechanisms underlying rat AhR activity need further investigation, which is significant for monitoring the endogenous AhR activation potential of new chemicals in humans.

Computational methods are important tools to explore the mechanisms of toxicities/activities at the molecular level [21,22,23], which is necessary to understand the molecular initiating events of various toxicities/activities. For example, molecular docking can predict the non-covalent binding of ligands and macromolecules [24], determine the best binding mode [25], and further provide bioaccumulation potential prediction [26], while molecular dynamics (MD) simulates the dynamic behavior of systems based on the integration of Newtonian equations [25]. They have already been applied to predict the mechanism of the AhR-mediated immune response [27] and the developmental toxicity [28] of some PACs. An immune response study [27] elucidated that PACs could form *π*-*π* interactions and hydrogen bonds with AhRs while inhibiting the transcription of CYP and ultimately inducing inflammatory cytokines. In addition, the docking results of 22 PACs with AhRs of three zebrafish isoforms and human AhRs, together with experimental results, proved their AhR activation and observable responses of developmental toxicity [28]. Another frequently used in silico method is developing a quantitative structure–activity relationship (QSAR) model, which employs a mathematical equation to quantitatively describe the relationship between the toxicity of chemicals and molecular descriptors [29]. The established QSAR models can provide estimated values of toxic indexes for organic chemicals with difficult-to-obtain experimental measurements, filling in the data gap. QSAR models have already been developed to predict the AhR activity [30] and the estrogen activity [31] of PAHs.

Thus, in this study, molecular docking and MD simulations were first performed to explore the binding affinity and interaction forces in PACs-rAhR complexes. Then, two QSAR models were developed to predict the rAhR activities and to further investigate the molecular mechanisms of rAhR-mediated activities according to the physicochemical properties of the employed descriptors. The expected results will help to better understand the rAhR activities of reactive PACs and provide an important basis for alerting structures to reduce health risks.

## 2. Results and Discussion

### 2.1. Molecular Docking and MD Simulation Results

Among the 50 candidate rAhR homology structures, the one with the lowest Molpdf value was selected as the optimal model structure because the smaller Molpdf score means higher similarity to the template PDB structures [32]. The constructed rAhR was further compared with the protein structures (3F1P, 3F1O, 3F1N, 3H7W, and 3H82) using the Rampage web servers (https://warwick.ac.uk/fac/sci/moac/people/students/peter cock/python/ramachandran/other/, accessed on 10 September 2024) and Protein Structure Analysis (ProSA) (https://prosa.services.came.sbg.ac.at/prosa.php, accessed on 10 September 2024). The results from the validation of the constructed 3D model using the Ramachandran plot from the MolProbity procedure showed that 89.5% of the total residues were in the favored region of the Ramachandran plot, and 97.1% were in the allowed region, with no bad bonds or angles in the protein geometry. This suggests that the protein backbone dihedral angles phi (Φ) and psi (Ψ) were reasonably accurate positions in the constructed 3D model (SI Figure S1). The results indicate that the Z-score (−1.94) for the 3D model was within the range of scores typically found for native proteins of similar size (SI Figure S2). Furthermore, the comparison of the homology model with five template models shows a slight difference in their backbone conformations, with the exception of the loop region (residues 317–345, SI Figure S3). Consequently, the results of the analysis of bond lengths, bond angles, Φ and Ψ angles from the Ramachandran plot, and total Cα atoms of structure concluded that the generated structural model was reliable for further studies.

**Figure 1:**
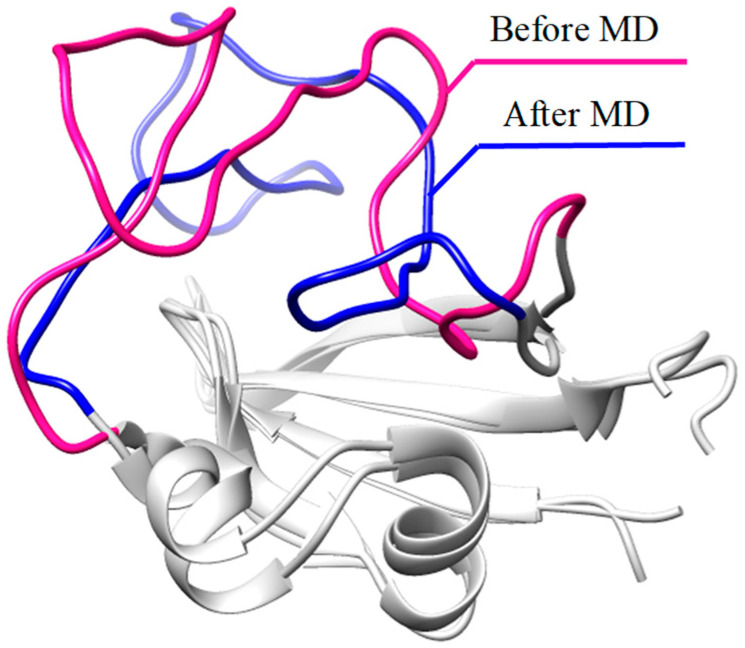
The superposition of the initial docking conformation with the representative MD conformation for the 7,12-benzo[a]anthraquinone-rAhR binding complex.

The docking results state that all PACs can bind tightly to rAhR, with the most negative energy scores ranging from −9.6 to −4.9 kcal/mol based on repeated docking (n > 10) (SI Table S1). It is found that the RMSD of template 3F1O and the rAhR homologous model is 2.3 Å, and the distance of the docking centers is 2.1 Å after aligning the two receptors to calibrate the docking protocols. Subsequently, the RMSD curves (SI Figure S4) demonstrate that the simulated conformations of the 15 binding complexes reach their respective equilibrium states. The RMSD values for all ligands are less than 2 Å, while the RMSD values for rAhR reach up to 8 Å, suggesting the high conformational change of rAhR during the MD simulations. In the case of the 7,12-benzo[a]anthraquinone-rAhR binding complex, the superposition between docking and MD configurations reveals the high fluctuation in the loop regions (residues 317–345, Figure 1), which thus leads to the high RMSF values (SI Figure S5).

The estimated Δ*G*_bind_ values range from −7.37 to −26.39 kcal/mol for the 15 complexes, as shown in Table S2. Δ*G*_bind_ exhibits a high correlation with van der Waals interaction *E*_vdw_ (Pearson relativity coefficient *R* = 0.98) and nonpolar solvation *G*_SA_ (*R* = 0.90) for the 15 complexes, revealing that *E*_vdw_ (−22.03 to −39.92 kcal/mol) and *G*_SA_ (−3.53 to −5.27 kcal/mol) play decisive effects on ligand binding. In contrast, the entropy effects (*TS*: −14.43 to −18.02 kcal/mol) provide the most adverse contributions to the binding of ligands. In addition, the electrostatic interaction energies (*E*_ele_: −0.45 to −3.16 kcal/mol), nonpolar solvation energies, and polar solvation energies (*G*_SA_: −3.53 to −5.27 kcal/mol, *G*_GB_: 2.67 to 4.99 kcal/mol) only make minor contributions.

As shown in Table S2, the binding affinity of ligand-rAhR complexes increases with the number of benzene rings, especially for PAHs and Cl-PAHs complexes. For instance, four PAHs with varying numbers of rings result in their binding affinities being the order of benzo[g]chrysene (−26.39 kcal/mol, five rings) > benzo[b]chrysene (−24.02 kcal/mol, five rings) > triphenylene (−19.35 kcal/mol, three rings) > fluorene (−15.24 kcal/mol, two rings). This trend has also been observed for benzo[g]chrysene and benzo[b]chrysene (five rings, Δ*G*_bind_ of −26.39 kcal/mol and −24.02 kcal/mol, respectively) vs. 2-methylchrysene (four rings, Δ*G*_bind_ of −19.10 kcal/mol). For Cl-PAHs, the Δ*G*_bind_ value of 9,10-dichlorophenanthrene with three rings is −13.56 kcal/mol, much larger than that of 7,12-dichlorobenz[a]anthracene with four rings (−25.06 kcal/mol), resulting in a weaker binding affinity. Similarly, the presence of halogen atoms may also enhance binding affinity, which can be confirmed by the comparison between 7-chlorobenz[a]anthracene and 7,12-dichlorobenz[a]anthracene (Δ*G*_bind_: −22.84 vs. −25.06 kcal/mol).

The amino acid residues with energy contributions of less than −1 kcal/mol are identified as key residues affecting ligand binding, as listed in SI Table S2. Among these, Pro295 gives important contributions to 10 PACs, including all O-PAHs, S-PAH, and Br-PAHs. Further analysis of specific PAH types revealed that Pro295 also has important contributions to the binding of chrysene derivatives and benz[a]anthracene derivatives. Another residue, Arg316, shows significant contributions to seven PACs, benzo[b]chrysene, 2-methylphenanthrene, 11-methylbenzo[a]pyrene, 4-nitropyrene, 7,11-dibromobenz[a]anthracene, and Cl-PAHs except for 7-chlorobenz[a]anthracene. Arg316 also plays an important role in the binding of all phenanthrene and pyrene derivatives.

**Figure 2:**
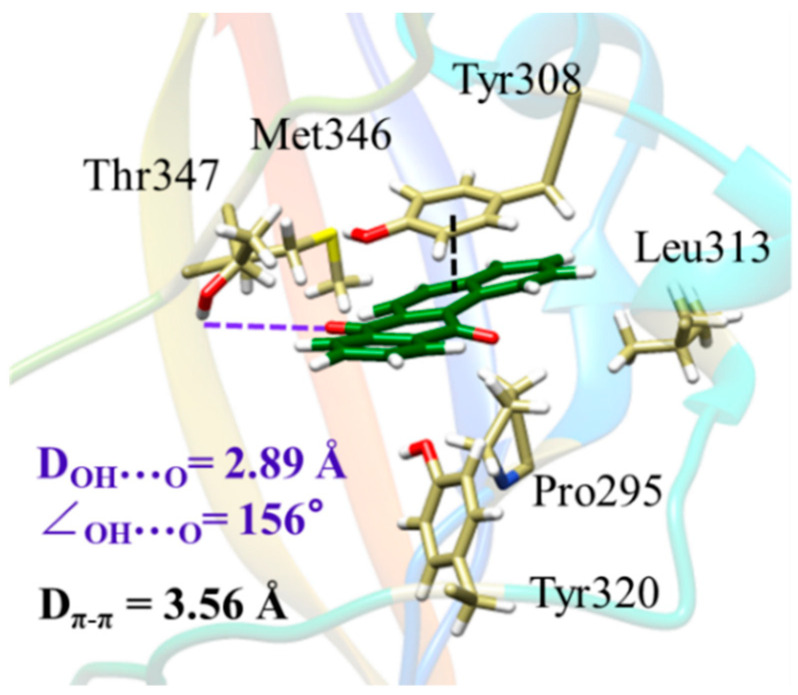
The hydrogen bond (dotted purple line) and *π*-*π* interaction (dark dotted line) between 7,12-benzo[a]anthraquinone and rAhR residues.

Except for van der Waals interactions, hydrogen bond interactions [33] can be observed in the binding conformations of 7H-benz[de]anthracen-7-one, 7,12-benzo[a]anthraquinone, and 4-nitropyrene. The NH group of Met346 can combine with the oxygen atom of 7H-benz[de]anthracen-7-one to form a hydrogen bond. His289 has a close affinity for 4-nitropyrene due to its NH group. The MD simulation shows that *π*-*π* interactions [34] occur in 7,12-benzo[a]anthraquinone and 9,10-dichlorophenanthrene complexes, and the benzene rings of Tyr308 and Phe349 approach the aromatic rings of the two compounds to form *π*-*π* interactions, respectively. The hydrogen bond and *π*-*π* interaction of 7,12-benzo[a]anthraquinone with rAhR residues are illustrated in Figure 2, and other chemicals are listed in SI Figure S6. Furthermore, the average distance between the oxygen atom and the hydroxyl hydrogen of the Thr347 residue is 2.89 Å, within the distance range for hydrogen bonds (generally taken as 3 Å). Meanwhile, the phenol ring of Tyr308 is approximately parallel to the ligand, with an average distance of 3.56 Å and an energy of −4.37 kcal/mol, producing *π*-*π* interaction to promote the binding of the ligand and rAhR.

Overall, the MD results of PACs-rAhR complexes indicate that the estimated Δ*G*_bind_ ranges from −7.37 to −26.39 kcal/mol and the numbers of benzene rings and halogen atom of PACs are the critical factors in binding affinities. Component analysis shows that van der Waals interactions serve asthe main driving force for the binding of ligands, with Pro295 and Arg316 identified as the most important residues in most complexes. Furthermore, hydrogen bonds and *π*-*π* interactions are formed between specific residues and PACs, contributing to their binding complexes. In the following, the QSAR models were further established for predictive purposes.

### 2.2. QSAR Models of IEQ for PAHs and Derivatives without Halogen

An optimal QSAR model for predicting log *IEQ* for 62 PACs without halogen was obtained via MLR analysis:

(1) $$\log\;\mathrm{IEQ}=10.29{\mathrm{SpMin}}_{2\mathrm{Bh}(m)}-9.03\;\times \;{\mathrm{HATS}}_{5p}\;-\;0.50\;\times \;\sigma -6.82\;\times \;{\mathrm{MATS}}_{5s}\;-\;5.58\;\times \;H_{6e}-20.30\;\times \;E_{2v}+2.43\;\times \;{\mathrm{SpMax}}_{8\mathrm{Bh}(i)}\;-\;12.83$$

**Figure 3:**
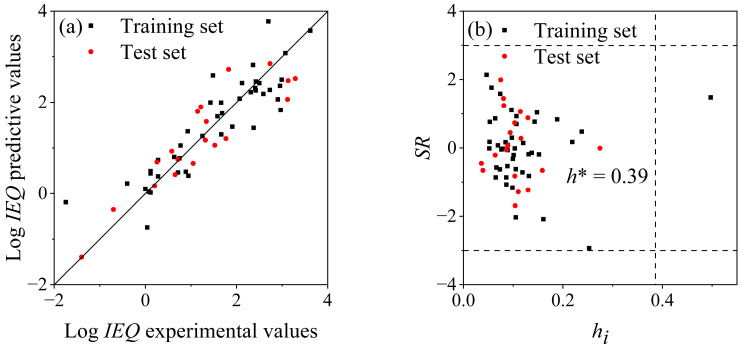
(**a**) Fitting plot of experimental and predictive log IEQ values by model (1), (**b**) Williams plot of model (1).

The selected descriptors are listed in Table 1, and the predicted log *IEQ* values and molecular descriptor values are listed in Table S3. The statistical parameters (Table 2) indicate a good fitting performance of the model. The results of the simulated external validation are also listed in Table 2. The *R*^2^ (0.80), *Q*^2^ (0.80), and *RMSE* (0.53) values of the re-established model, based on the training set (70% log *IEQ* experimental values), are the same as those of model (1), indicating the intrinsic correlation between the descriptors and the log *IEQ* values. The statistic parameters of the test set (*R*^2^ = 0.79, *Q*^2^ = 0.79 and *RMSE* = 0.57) validate the good predictive ability of the QSAR model. As shown in Figure 3a, the predicted log *IEQ* values are consistent with the experimental values for both the training and test sets.

Moreover, *Q*^2^_F1_ is 0.83, *Q*^2^_F2_ is 0.82, *Q*^2^_F3_ is 0.80, and *CCC* is 0.86, all of which meet the acceptable criteria (*Q*^2^_Fn_ > 0.70, *CCC* > 0.85). Leave-one-out cross validation results (*Q*^2^_CV_ is 0.85 and *RMSE*_CV_ is 0.64) reflect that the model demonstrates good robustness. The application domain (Figure 3b) illustrates that the leverage of fluorine (0.50) is larger than the threshold value (h* = 0.39), but its |*SR*| is less than 3, indicating that model (1) has good extrapolation ability.

The descriptors *SpMin*_2Bh(m)_ and *SpMax*_8Bh(i)_ belong to Burden matrix descriptors relating to molecular mass [35] and molecular ionization potential [36], respectively. The positive correlation between *SpMin*_2Bh(m)_ and *SpMax*_8Bh(i)_ with log *IEQ* indicates that greater molecular mass and stronger ionization potential can cause lower rAhR activity. *HATS*_5p_ stands for molecular polarizability [37], and larger *HATS*_5p_ can lead to increased rAhR activity. Softness (*σ*), which reflects electron activity and molecular reactivity [38], is negatively associated with log *IEQ*, indicating that rAhR activity increases with chemical reactivity. *MATS*_5s_ is related to the electron distribution within molecules [39,40], and a larger value results in a larger probability of the molecule binding to rAhR, further yielding stronger rAhR activity. *H*_6e_ characterizes the electronegativity of hydrogen atoms [41], and a smaller *H*_6e_ value indicates that the chemical is less likely to bind with amino acids, leading to weaker rAhR activity. *E*_2v_ is a WHIM index weighted by atomic van der Waals volumes, and a larger *E*_2v_ value can cause a larger rAhR activity, as shown by the negative correlation in the model [42,43]. In conclusion, the rAhR activity of 62 PACs without halogen is mainly governed by molecular mass, ionization potential, polarizability, electronegativity, and van der Waals volumes.

### 2.3. QSAR Models of Log%-TCDD-Max for Cl-PAHs and Br-PAHs

The QSAR model of log%-TCDD-*max* for 21 halogenated PAHs was successfully constructed, as shown in Equation (2):

(2) $$\log\%-\mathrm{TCDD}-\max=-0.80\;\times \;\mathrm{HGM}-33.75\;\times \;E_{\mathrm{EHOMO}}+31.71\;\times \;{\mathrm{ATSC}}_{1e}-3.34$$

The experimental and predictive log%-TCDD-*max* values, description, and calculated values of the selected descriptors are provided in Table 3 and Table S4, respectively. The statistical parameters (listed in Table 4) reveal a good regression performance of model (2) (*R*^2^ = 0.89, *Q*^2^ = 0.89, and *RMSE* = 0.21).

**Figure 4:**
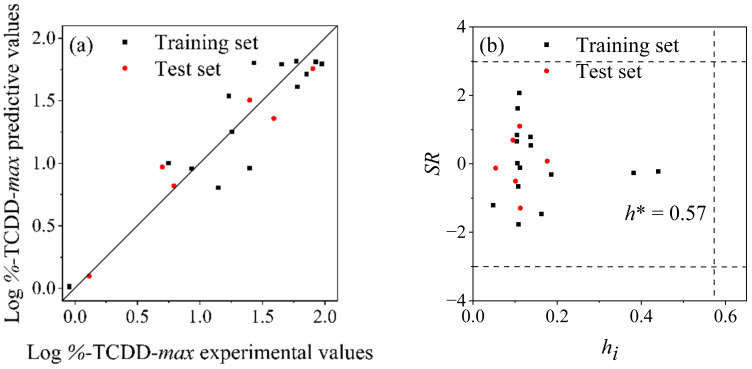
(**a**) Fitting plot of experimental and predictive log%-TCDD-*max* values using model (2), (**b**) Williams plot of model (2).

The results of the simulated external validation (*R*^2^ = 0.88, *Q*^2^ = 0.88, and *RMSE* = 0.23 for the training set and *R*^2^ = 0.93, *Q*^2^ = 0.92, and *RMSE* = 0.19 for the test set, respectively) and the favorable consistency of experimental and predictive log%-TCDD-*max* values for both the training set and test set, as shown in Figure 4a, as well as the statistical parameters *Q*^2^_F1_ (0.93), *Q*^2^_F2_ (0.93), *Q*^2^_F3_ (0.93), and *CCC* (0.96), prove the good predictive ability of model (2). The internal validation results, *Q*^2^_CV_ = 0.92 and *RMSE*_CV_ = 0.24, indicate the preferable robustness of the model. The Williams plot (Figure 4b) illustrates no outliers.

*HGM* is a GETAWAY descriptor that inversely relates to molecular size, decreasing with the increase in atom number in the molecule [44,45]. The negative coefficient indicates that increasing the complexity of halogenated PAHs can increase its rAhR activity. It is interesting that *E*_HOMO_ was selected in the model instead of *E*_LUMO_, which reflects the molecular nucleophilicity [46]. Generally, halogenated PAHs act as electrophilic compounds to react with the amino acid residues of rAhR as described above. Thus, a higher *E*_HOMO_ value may impede such interactions and subsequently result in lower rAhR activity. *ATSC*_1e_ is related to Sanderson electronegativity [47], the molecular equalized electronegativity when the electronegativities of the constituent atoms are equal [48]. The positive regression coefficient between *ATSC*_1e_ and log%-TCDD-*max* means that a higher Sanderson electronegativity of a chemical accounts for higher rAhR activity. Therefore, the rAhR activity of halogenated PAHs is greatly influenced by molecular size, nucleophilicity, and Sanderson electronegativity.

The results of MD simulation indicate that van der Waals interactions account for the major contribution in forming complexes of PACs and rAhR. Then, the developed QSAR model of log *IEQ* for 62 PACs without halogen atoms suggests that polarizability is a crucial factor in determining the rAhR activity of these chemicals. The expansion of the mean interatomic distance caused by nuclear quantum effects can enhance non-covalent van der Waals interactions and increase molecular polarizability [49]. Thus, the chemicals with a large polarizability may lead to a strong van der Waals interaction and promote the binding affinity of PACs, ultimately increasing rAhR activity.

In fact, molecular polarizability is an important parameter related to the biological activities of organic chemicals. It has been proved to play significant roles in the binding to estrogen receptors [31] and O-PAHs binding to DNA [50]. A previous QSAR model for AhR-mediated luciferase activity of PAHs [30] suggested that molecular polarizability and aromaticity are the main factors that enhance the partition of PAHs within the cell membrane to bind with AhR and result in high AhR activities. Similarly, our QSAR models also show the importance of molecular polarizability in relation to the rAhR activity of PACs. Therefore, molecular polarizability is closely associated with the toxic mechanisms of organic chemicals, deserving more attention in future research.

In this work, the adverse impact of specific receptors with the largest dataset of experimental data of PACs was used to explore molecular mechanisms and modes of action with rAhR. In virtue of the QSAR models, we expect to be able to classify or predict the luciferase activity of 350 PACs that have been discovered [51], providing valuable information for toxicity and risk assessment. Scaling these models to other enzymes would be another step in generalizing these models for application in predictive models. Then, information regarding abnormal behaviors or activity expression could be related to toxicodynamic information of the chemical obtained from in vitro test systems to perform a comprehensive risk assessment of a new PAC.

## 3. Material and Methods

### 3.1. Dataset

The relative rAhR induction equivalents (*IEQ*, pg/g) of 62 PACs without halogen elements (35 PAHs, 20 M-PAHs, 2 O-PAHs, 3 S-PAHs, and 2 N-PAHs) relative to 2, 3, 7, 8-tetrachlodizenzo-*p*-dioxin (TCDD) were summarized by Lenka Pálková et al. [52]. They were measured by the chemical-activated luciferase expression (CALUX) assay using rat hepatoma H4IIE cells stably transfected with an rAhR-inducible luciferase reporter gene. The *IEQ*s were related to the chemical concentrations causing 25% maximal luciferase induction, which shows a negative relationship with rAhR activity. For 21 halogenated PACs, including 15 Cl-PAHs and 6 Br-PAHs, the observed maximum response was determined using a rat H4IIE-*luc* in vitro bioassay and expressed as a percentage of the mean maximum response of the TCDD standard (%-TCDD-*max*) [53]. Large %-TCDD-*max* values for the halogenated PACs suggested the high potency of these chemicals to induce rAhR-mediated activities. In this work, the logarithmic values (log *IEQ* and log%-TCDD-*max*) were utilized, as listed in the Supporting Information (SI) Table S1.

### 3.2. Homology Modeling, Molecular Docking, and MD Simulations

Since no available crystal structure of rAhR has been reported to date, we thus constructed the relevant LBD via homology modeling, with details provided in the SI.

AutoDock Tools 1.5.6 was used to identify the potential ligand binding site [54] (SI Figure S7) in the constructed structure of rAhR based on the ligand-bound 3F1O [10]. The AutoDock Vina program [24] was used to perform docking simulations of a total of 83 PACs within rAhR to generate the ligand–receptor binding complexes. The complexes with the most negative binding energy scores were employed as the initial binding configurations for the subsequent MD simulations.

In order to accurately evaluate binding interactions, MD simulations were further performed using the Amber12 program [55,56] for a total of 15 typical docked complexes, including PAHs (fluorene, triphenylene, benzo[b]chrysene, and benzo[g]chrysene), M-PAHs (2-methylphenanthrene, 2-methylchrysene, and 11-methylbenzo[a]pyrene), O-PAHs (7H-benz[de]anthracen-7-one and 7,12-benzo[a]anthraquinone), S-PAH (Benzo[b]naphtho [2,1-d]thiophene), N-PAH (4-nitropyrene), Cl-PAHs (9,10-dichlorophenanthrene, 7-chlorobenz[a]anthracene, and 7,12-dichlorobenz[a]anthracene), and Br-PAH (7,11-dibromobenz[a]anthracene) with rAhR as the initial binding conformations. The ff14SB force field [57] was used to describe the system’s topology for the binding complexes. During the MD simulations, the protonation states of the polar receptor residues were first determined based on the PDB2PQR service [58]. Concretely, His289, His330, and His335 residues were singly N*_δ_*-protonated, the His355 residue was set to be N*_ε_*-protonated, while His324 was fully protonated at both the N*_δ_* and N*_ε_* atoms. Then, each solvation model was sequentially subjected to the steepest descent minimization for 4000 steps, gradual heating from 0 to 300 K over 500 ps, and constant pressure equilibration for 1000 ps at 300 K. Subsequent production MD simulations with varying time scales (70–200 ns) were carried out for the binding complexes to obtain the equilibrium conformations. Based on the MD trajectories, the root-mean-square deviations (RMSD, Å) [59] and root-mean-square fluctuations (RMSF, Å) [22] of the ligand and receptor were used to evaluate the binding conformational stability and protein flexibility, respectively. Through cluster analyses of the equilibrium configurations [60], the class with the largest populated configurations was selected. The 200 snapshots from the largest cluster were extracted to calculate the binding free energy (Δ*G*_bind_) of the ligand in rAhR and to identify the key residues that significantly contribute to ligand binding. Δ*G*_bind_ was estimated according to the Molecular Mechanics/Generalized Born Surface Area (MM/GBSA) [61] method, and the corresponding formulas were shown in SI.

### 3.3. QSAR Model

According to previous experience [62,63,64], Dragon descriptors and electronic descriptors [65] were calculated to characterize molecular structure properties. Then, a multiple linear regression (MLR) strategy was used to select descriptors and develop QSAR models using IBM SPSS Statistics 21 software [66]. Simulated external validation and leave-one-out cross-validation were employed to test the predictive ability and robustness of the developed models. The details of descriptor calculation, statistical analysis, and model validation are shown in the SI.

## 4. Conclusions

Molecular docking and MD simulations show that van der Waals forces are the major driving force in PACs-rAhR complexes, and Pro295 and Arg316 are the important amino acid residues in ligand binding. For 62 PACs without halogen atoms, the rAhR activity is mainly influenced by the molecular mass, ionization potential, polarizability, electronegativity, and van der Waals volumes. For 21 halo-PACs, the rAhR activity is related to molecular size, nucleophilicity, and Sanderson electronegativity. The developed models have good predictive ability, robustness, and extrapolation ability; thus, they can be used to predict the rAhR activity of other reactive PACs within the application domain.

## Figures and Tables

**Table 1:** Physical–chemical meanings of the descriptors used in QSAR models.

Descriptor	Physical-Chemical Meanings
*SpMin*_2Bh(m)_	Smallest eigenvalue n.2 of Burden matrix weighted by mass Burden eigenvalues
*HATS*_5p_	Leverage-weighted autocorrelation of lag 5 weighted by polarizability GETAWAY descriptors
*σ*	Softness
*MATS*_5s_	Moran autocorrelation of lag 5 weighted by I-state 2D autocorrelations
*H*_6e_	H autocorrelation of lag 6 weighted by Sanderson electronegativity GETAWAY descriptors
*E*_2v_	The 2nd component accessibility directional WHIM index weighted by van der Waals volume WHIM descriptor
*SpMax*_8Bh(i)_	Largest eigenvalue n. 8 of Burden matrix weighted by ionization potential Burden eigenvalues

**Table 2:** Statistical parameters of log *IEQ* models for 62 PACs without halogen and simulated external validation results.

	*N*	*R* ^2^	*Q* ^2^	*RMSE*	*BIAS*	*MAE*	*MPE*	*MNE*
Model (1)	62	0.80	0.80	0.53	0.00	0.40	1.55	−1.14
Training set	43	0.80	0.80	0.53	0.00	0.40	1.40	−1.09
Test set	19	0.79	0.79	0.57	−0.03	0.46	1.02	−1.08

**Table 3:** Physical–chemical meanings of the descriptors.

Descriptor	Physical–Chemical Meanings
*HGM*	Geometric mean on the leverage magnitude GETAWAY descriptors
*E*_HOMO_	The energy of the highest occupied molecular orbital
*ATSC*_1e_	Centred Broto–Moreau autocorrelation of lag 1 weighted by Sanderson electronegativity 2D autocorrelations

**Table 4:** Statistical parameters of log%-TCDD-*max* models for 21 halogenated PAHs and simulated external validation results.

	*N*	*R* ^2^	*Q* ^2^	*RMSE*	*BIAS*	*MAE*	*MPE*	*MNE*
Model (2)	21	0.89	0.89	0.21	−0.00	0.16	0.37	−0.44
Training set	15	0.88	0.88	0.23	−0.00	0.18	0.36	−0.44
Test set	6	0.93	0.92	0.19	0.02	0.15	0.27	−0.25

## Data Availability

The data supporting the reported results can be found in Supplementary Materials Tables S1–S4.

## Supplementary Materials

The following supporting information can be downloaded at: https://www.mdpi.com/article/10.3390/molecules29194619/s1. Figure S1: Ramachandran plot image of rAhR homology structure based on 3F1O which has the native ligand. Figure S2: The ProSA analyses of the generated rAhR LBD structure model. Figure S3: Structural aligning the structures of the homology rAhR model (pink loop) and five template models (chain A of 3F1P, 3F1O, 3F1N, 3H7W and chain B of 3H82) on their C*α* atoms. Figure S4: The root-mean-square deviation (RMSD, Å) of 15 types of PACs and rAhR. Figure S5: The root-mean-square fluctuations (RMSF, Å) of 15 types rAhR bound with PACs. Figure S6: The hydrogen bond and *π*-*π* interaction diagram of complexes which 7H-benz[de]anthracen-7-one, 4-nitropyrene and 9,10-dichlorophenanthrene were bound with rAhR. Figure S7: The model of rat aryl hydrocarbon receptor (rAhR), the dark frame is binding site; Table S1: Autodock vina scores of 83 PACs with rAhR. Table S2: The amino acid residues with energy contributions of 15 types of PACs. Table S3: The experimental and predictive log *IEQ* values and selected molecular descriptors values in model (1). Table S4: The experimental and predictive log %-TCDD-*max* values and selected molecular descriptors values in model (2). Refs [67,68,69,70,71,72,73,74,75,76,77,78,79,80,81] are cited in Supplementary Materials.

## References

[1] Darryl P.A., David J.S., Daniel C.M. (1996). The effects of near ultraviolet radiation on the toxic effects of polycyclic aromatic hydrocarbons in animals and plants: A review. Ecotoxicol. Environ. Saf..

[2] Davie-Martin C.L., Stratton K.G., Teeguarden J.G., Waters K.M., Simonich S.L.M. (2017). Implications of bioremediation of polycyclic aromatic hydrocarbon-contaminated soils for human health and cancer risk. Environ. Sci. Technol..

[3] Jiang L., Ma X., Wang Y., Gao W., Liao C., Gong Y., Jiang G. (2022). Land-ocean exchange mechanism of chlorinated paraffins and polycyclic aromatic hydrocarbons with diverse sources in a coastal zone boundary area, North China: The role of regional atmospheric transmission. Environ. Sci. Technol..

[4] Krzyszczak A., Czech B. (2021). Occurrence and toxicity of polycyclic aromatic hydrocarbons derivatives in environmental matrices. Sci. Total Environ..

[5] Lundstedt S., Bandowe B.A.M., Wilcke W., Boll E., Christensen J.H., Vila J., Grifoll M., Faure P., Biache C., Lorgeoux C. (2014). First intercomparison study on the analysis of oxygenated polycyclic aromatic hydrocarbons (oxy-PAHs) and nitrogen heterocyclic polycyclic aromatic compounds (N-PACs) in contaminated soil. TrAC Trends Anal. Chem..

[6] Abbas I., Badran G., Verdin A., Ledoux F., Roumié M., Courcot D., Garçon G. (2018). Polycyclic aromatic hydrocarbon derivatives in airborne particulate matter: Sources, analysis and toxicity. Environ. Chem. Lett..

[7] Idowu O., Semple K.T., Ramadass K., O’connor W., Hansbro P., Thavamani P. (2019). Beyond the obvious: Environmental health implications of polar polycyclic aromatic hydrocarbons. Environ. Int..

[8] Marvin C.H., Tomy G.T., Thomas P.J., Holloway A.C., Sandau C.D., Idowu I., Xia Z. (2020). Considerations for prioritization of polycyclic aromatic compounds as environmental contaminants. Environ. Sci. Technol..

[9] Hirano M., Hwang J.H., Park H.J., Bak S.M., Iwata H., Kim E.Y. (2015). In silico analysis of the interaction of avian aryl hydrocarbon receptors and dioxins to decipher isoform-, ligand-, and species-specific activations. Environ. Sci. Technol..

[10] Nuti R., Gargaro M., Matino D., Dolciami D., Grohmann U., Puccetti P., Fallarino F., Macchiarulo A. (2014). Ligand binding and functional selectivity of L-tryptophan metabolites at the mouse aryl hydrocarbon receptor (mAhR). J. Chem. Inf. Model..

[11] Misaki K., Kawami H., Tanaka T., Handa H., Nakamura M., Matsui S., Matsuda T. (2007). Aryl hydrocarbon receptor ligand activity of polycyclic aromatic ketones and polycyclic aromatic quinones. Environ. Toxicol. Chem..

[12] Hoang A.Q., Suzuki G., Michinaka C., Tue N.M., Tuyen L.H., Tu M.B., Takahashi S. (2021). Characterization of unsubstituted and methylated polycyclic aromatic hydrocarbons in settled dust: Combination of instrumental analysis and in vitro reporter gene assays and implications for cancer risk assessment. Sci. Total Environ..

[13] Lam M.M., Bulow R., Engwall M., Giesy J.P., Larsson M. (2018). Methylated PACs are more potent than their parent compounds: A study of aryl hydrocarbon receptor-mediated activity, degradability, and mixture interactions in the H4IIE-*luc* assay. Environ. Toxicol. Chem..

[14] Larsson M., Hagberg J., Giesy J.P., Engwall M. (2014). Time-dependent relative potency factors for polycyclic aromatic hydrocarbons and their derivatives in the H4IIE-*luc* bioassay. Environ. Toxicol. Chem..

[15] Kanae B., Hidetaka T., Go S., Ning T., Kazuichi H. (2009). Evaluation of toxic activities of polycyclic aromatic hydrocarbon derivatives using in vitro bioassays. J. Health Sci..

[16] Larsson M., Giesy J.P., Engwall M. (2014). AhR-mediated activities of polycyclic aromatic compound (PAC) mixtures are predictable by the concept of concentration addition. Environ. Int..

[17] Lubcke-Von Varel U., Machala M., Ciganek M., Neca J., Pencikova K., Palkova L., Vondracek J., Loffler I., Streck G., Reifferscheid G. (2011). Polar compounds dominate in vitro effects of sediment extracts. Environ. Sci. Technol..

[18] Sonneveld E., Jonas A., Meijer O.C., Brouwer A., Van Der Burg B. (2007). Glucocorticoid-enhanced expression of dioxin target genes through regulation of the rat aryl hydrocarbon receptor. Toxicol. Sci..

[19] Brown M.R., Garside H., Thompson E., Atwal S., Bean C., Goodall T., Sullivan M., Graham M.J. (2017). From the cover: Development and application of a dual rat and human ahr activation assay. Toxicol. Sci..

[20] Ohura T., Morita M., Makino M., Amagai T., Shimoi K. (2007). Aryl hydrocarbon receptor-mediated effects of chlorinated polycyclic aromatic hydrocarbons. Chem. Res. Toxicol..

[21] Chai L., Zhang H., Song R., Yang H., Yu H., Paneth P., Kepp K.P., Akamatsu M., Ji L. (2021). Precision biotransformation of emerging pollutants by human cytochrome p450 using computational-experimental synergy: A case study of tris(1,3-dichloro-2-propyl) phosphate. Environ. Sci. Technol..

[22] Ma G., Yu H., Han C., Jia Y., Wei X., Wang Z. (2020). Binding and metabolism of brominated flame retardant beta-1,2-dibromo-4-(1,2-dibromoethyl)cyclohexane in human microsomal P450 enzymes: Insights from computational studies. Chem. Res. Toxicol..

[23] Morris C.J., Corte D.D. (2021). Using molecular docking and molecular dynamics to investigate protein-ligand interactions. Mod. Phys. Lett. B.

[24] Trott O., Olson A.J. (2010). AutoDock Vina: Improving the speed and accuracy of docking with a new scoring function, efficient optimization, and multithreading. J. Comput. Chem..

[25] Salmaso V., Moro S. (2018). Bridging molecular docking to molecular dynamics in exploring ligand-protein recognition process: An overview. Front. Pharmacol..

[26] Ng C.A., Hungerbuehler K. (2015). Exploring the use of molecular docking to identify bioaccumulative perfluorinated alkyl acids (PFAAs). Environ. Sci. Technol..

[27] Wang C., Yang J., Zhu L., Yan L., Lu D., Zhang Q., Zhao M., Li Z. (2017). Never deem lightly the “less harmful” low-molecular-weight PAH, NPAH, and OPAH-disturbance of the immune response at real environmental levels. Chemosphere.

[28] Chlebowski A.C., Garcia G.R., La Du J.K., Bisson W.H., Truong L., Massey Simonich S.L., Tanguay R.L. (2017). Mechanistic investigations into the developmental toxicity of nitrated and heterocyclic PAHs. Toxicol. Sci..

[29] EPA (2014). Guidance Document on the Validation of (Quantitative) Structure-Activity Relationship [(Q)SAR] Models. OECD Environment Health and Safety Publications Series on Testing and Assessment, 2007. Guidance Document on the Validation of (Quantitative) Structure-Activity Relationship [(Q)SAR] Models.

[30] Wang Y., Yang X., Zhang S., Guo T.L., Zhao B., Du Q., Chen J. (2021). Polarizability and aromaticity index govern AhR-mediated potencies of PAHs: A QSAR with consideration of freely dissolved concentrations. Chemosphere.

[31] Fei L., Huifeng W., Lianzhen L., Xuehua L., Jianmin Z., Peijnenburg W.J.G.M. (2012). Docking and QSAR study on the binding interactions between polycyclic aromatic hydrocarbons and estrogen receptor. Ecotoxicol. Environ. Saf..

[32] Miyagi S., Sawamura S., Yoshikawa E., Dedachi K., Itoh S., Ishihara-Sugano M., Kurita N. (2012). Ab initio fragment molecular orbital calculations on specific interactions between aryl hydrocarbon receptor and dioxin. Int. J. Quantum Chem..

[33] Steiner T. (2002). The hydrogen bond in the solid state. Angew. Chem. Int. Ed..

[34] Lee E.C., Kim D., Jurečka P., Tarakeshwar P., Hobza P., Kim K.S. (2007). Understanding of assembly phenomena by aromatic-aromatic interactions-benzene dimer and the substituted systems. J. Phys. Chem. A.

[35] Borges De Melo E., Ataide Martins J.P., Marinho Jorge T.C., Friozi M.C., Castro Ferreira M.M. (2010). Multivariate QSAR study on the antimutagenic activity of flavonoids against 3-NFA on Salmonella typhimurium TA98. Eur. J. Med. Chem..

[36] Todeschini R., Consonni V. (2000). Handbook of Molecular Descriptors.

[37] Luo J., Lai T., Guo T., Chen F., Zhang L., Ding W., Zhang Y. (2018). Synthesis and acaricidal activities of scopoletin phenolic ether derivatives: QSAR, molecular docking study and in silico adme predictions. Molecules.

[38] Mali S.N., Pandey A. (2021). Molecular modeling studies on 2,4-disubstituted imidazopyridines as anti-malarials: Atom-based 3D-QSAR, molecular docking, virtual screening, in-silico admet and theoretical analysis. J. Comput. Biophys. Chem..

[39] Lowell H.H., Brian M., Lemont B.K. (1991). The Electrotopological State An Atom Index for QSAR. Quant. Struct.-Act. Relat..

[40] Lowell H.H. (1995). Electrotopological state indices for atom types a novel combination of electronic topological, and valence state information. J. Chem. Inf. Model..

[41] Kaya S., Kaya C. (2015). A new equation based on ionization energies and electron affinities of atoms for calculating of group electronegativity. Comput. Theor. Chem..

[42] Cruz-Monteagudo M., Borges F., Perez Gonzalez M., Cordeiro M.N. (2007). Computational modeling tools for the design of potent antimalarial bisbenzamidines: Overcoming the antimalarial potential of pentamidine. Bioorg. Med. Chem..

[43] Comelli N.C., Duchowicz P.R., Castro E.A. (2014). QSAR models for thiophene and imidazopyridine derivatives inhibitors of the Polo-Like Kinase 1. J. Pharm. Sci..

[44] Li J., Du J., Xi L., Liu H., Yao X., Liu M. (2009). Validated quantitative structure-activity relationship analysis of a series of 2-aminothiazole based p56Lck inhibitors. Anal. Chim. Acta.

[45] González M.P., Terán C., Teijeira M., González-Moa M.J. (2005). GETAWAY descriptors to predicting A2A adenosine receptors agonists. J. Med. Chem..

[46] Wei X., Yang M., Zhu Q., Wagner E.D., Plewa M.J. (2020). Comparative quantitative toxicology and QSAR modeling of the haloacetonitriles: Forcing agents of water disinfection byproduct toxicity. Environ. Sci. Technol..

[47] Marcin G., Szewczyk-Golec K., Pluskota R., Koba M., Mądra-Gackowska K., Woźniak A. (2022). Application of multivariate adaptive regression splines (MARSplines) for predicting antitumor activity of anthrapyrazole derivatives. Int. J. Mol. Sci..

[48] Bultinck P., Vanholme R., Popelier PL A., De Proft F., Geerlings P. (2004). High-speed calculation of AIM charges through the electronegativity equalization method. J. Phys. Chem. A.

[49] Huziel E.S., Valentin V.-G., Stefan C., Klaus-Robert M., Alexandre T. (2021). Dynamical strengthening of covalent and non-covalent molecular interactions by nuclear quantum effects at finite temperature. Nat. Commun..

[50] Li F., Li X., Liu X., Zhang L., You L., Zhao J., Wu H. (2011). Noncovalent interactions between hydroxylated polycyclic aromatic hydrocarbon and DNA: Molecular docking and QSAR study. Environ. Toxicol. Pharmacol..

[51] Li T., Su W., Zhong L., Liang W., Feng X., Zhu B., Ruan T., Jiang G. (2023). An integrated workflow assisted by in silico predictions to expand the list of priority polycyclic aromatic compounds. Environ. Sci. Technol..

[52] Pálková L., Vondráček J., Trilecová L., Ciganek M., Pěnčíková K., Neča J., Milcová A., Topinka J., Machala M. (2015). The aryl hydrocarbon receptor-mediated and genotoxic effects of fractionated extract of standard reference diesel exhaust particle material in pulmonary, liver and prostate cells. Toxicol. In Vitro.

[53] Yuichi H., Jong Seong K., Eric B.H., John P.G., Takeshi O., Kurunthachalam K. (2009). Relative potencies of individual chlorinated and brominated polycyclic aromatic hydrocarbons for induction of aryl hydrocarbon receptor-mediated responses. Environ. Sci. Technol..

[54] Seeliger D., De Groot B.L. (2010). Ligand docking and binding site analysis with PyMOL and Autodock/Vina. J. Comput. Aided. Mol. Des..

[55] Junmei W., Romain M.W., James W.C., Peter A.K., David A.C. (2004). Development and testing of a general amber force field. J. Chem. Theory Comput..

[56] Case D.A., Cheatham T.E., Darden T., Gohlke H., Luo R., Merz K.M., Onufriev A., Simmerling C., Wang B., Woods R.J. (2005). The Amber biomolecular simulation programs. J. Comput. Chem..

[57] Maier J.A., Martinez C., Kasavajhala K., Wickstrom L., Hauser K.E., Simmerling C. (2015). ff14SB: Improving the accuracy of protein side chain and backbone parameters from ff99SB. J. Chem. Theory Comput..

[58] Dolinsky T.J., Nielsen J.E., Mccammon J.A., Baker N.A. (2004). PDB2PQR: An automated pipeline for the setup of Poisson-Boltzmann electrostatics calculations. Nucleic Acids Res..

[59] Badry D.B., Maxim T., Ruben A., Charles L.B. (2003). Comparative study of several algorithms for flexible ligand docking. J. Comput. Aid. Mol. Des..

[60] Lei H., Wu C., Liu H., Duan Y. (2007). Folding free-energy landscape of villin headpiece subdomain from molecular dynamics simulations. Proc. Natl. Acad. Sci. USA.

[61] Xu L., Sun H., Li Y., Wang J., Hou T. (2013). Assessing the performance of MM/PBSA and MM/GBSA methods. 3. The impact of force fields and ligand charge models. J. Phys. Chem. B.

[62] Wei X., Yuan Q., Serge B., Xu T., Ma G., Yu H. (2017). In silico investigation of gas/particle partitioning equilibrium of polybrominated diphenyl ethers (PBDEs). Chemosphere.

[63] Fu Z., Chen J., Li X., Wang Y., Yu H. (2016). Comparison of prediction methods for octanol-air partition coefficients of diverse organic compounds. Chemosphere.

[64] Liu S., Jin L., Yu H., Lv L., Chen C.E., Ying G.G. (2020). Understanding and predicting the diffusivity of organic chemicals for diffusive gradients in thin-films using a QSPR model. Sci. Total Environ..

[65] Parthasarathi R., Dhawan A. (2018). In silico approaches for predictive toxicology. Vitro Toxicol.

[66] Hashemianzadeh M., Safarpour M.A., Gholamjani-Moghaddam K., Mehdipour A.R. (2008). DFT-based QSAR study of valproic acid and its derivatives. QSAR Comb. Sci..

[67] Andra´ s F., Andrej S.a. (2003). Modeller-generation and refinement of homology-based protein structure models. Methods Enzymol..

[68] Chirico N., Gramatica P. (2012). Real external predictivity of qsar models. Part 2. New intercomparable thresholds for different validation criteria and the need for scatter plot inspection. J. Chem. Inf. Model..

[69] United States Environmental Protection Agency (2012). Estimation Programs Interface Suite[TM] for Microsoft Windows.

[70] Erbel P.J., Card P.B., Karakuzu O., Bruick R.K., Gardner K.H. (2003). Structural basis for PAS domain heterodimerization in the basic helix-loop-helix-PAS transcription factor hypoxia-inducible factor. Proc. Natl. Acad. Sci. USA.

[71] Eriksson L., Jaworska J., Worth A.P., Cronin M.T.D., McDowell R.M., Gramatica P. (2003). Methods for reliability and uncertainty assessment and for applicability evaluations of classification- and regression-based QSARs. Environ. Health Perspect..

[72] Faber S.C., Giani Tagliabue S., Bonati L., Denison M.S. (2020). The cellular and molecular determinants of naphthoquinone-dependent activation of the aryl hydrocarbon receptor. Int. J. Mol. Sci..

[73] Frisch M.J., Trucks G.W., Schlegel H.B., Scuseria G.E., Robb M.A., Cheeseman J.R., Scalmani G., Barone V., Mennucci B., Petersson G.A. (2009). Gaussian 09.

[74] Mark H., Eibe F., Geoffrey H., Bernhard P., Peter R., Ian H.W. (2009). The Weka data mining software: An update. SIGKDD Explor..

[75] Medicine N.L.O. UniProtKB/Swiss-Prot, United States Government. https://www.expasy.org/resources/uniprotkb-swiss-prot.

[76] Mohaddeseh B., Ibrahim T., Mehrdad M., Gholamreza A., Andrew J.E. (2012). Comparative modeling of CCRL1, a key protein in masked immune diseases and virtual screening for finding inhibitor of this protein. Bioinformation.

[77] Motto I., Bordogna A., Soshilov A.A., Denison M.S., Bonati L. (2011). New aryl hydrocarbon receptor homology model targeted to improve docking reliability. J. Chem. Inf. Model..

[78] Taklete S. Dragon for Windows (Software for Molecular Descriptor Calculations), Version 6. http://www.talete.mi.it/products/dragon_description.htm.

[79] Saavedra L.M., Duchowicz P.R. (2021). Predicting zebrafish (*Danio rerio*) embryo developmental toxicity through a non-conformational QSAR approach. Sci. Total Environ..

[80] Todeschini R., Ballabio D., Grisoni F. (2016). Beware of unreliable Q2! A comparative study of regression metrics for predictivity assessment of QSAR models. J. Chem. Inf. Model..

[81] Zhao Y., Truhlar D.G. (2008). The M06 suite of density functionals for main group thermochemistry, thermochemical kinetics, noncovalent interactions, excited states, and transition elements two new functionals. Theor. Chem. Acc..

