# Supplementary material for: Molecular Interactions Governing the Rat Aryl Hydrocarbon Receptor Activities of Polycyclic Aromatic Compounds and Predictive Model Development
Source: Molecules. 2024 Sep 29;29(19):4619. doi: 10.3390/molecules29194619 (PMC11477797; doi:10.3390/molecules29194619)
Supplement: Supplementary file 1 [file molecules-29-04619-s001.zip › molecules-3155589-supplementary.pdf]

Supporting Information

Molecular interactions governing the rat aryl hydrocarbon receptor activities of polycyclic aromatic compounds and predictive model development

Lingmin Jin[a,b†], Bangyu Chena[†], Guangcai Ma[a], Xiaoxuan Wei[a], Haiying Yu[a*]

[a] College of Geography and Environmental Sciences, Zhejiang Normal University, Jinhua 321004, China

[b] School of Environment Science and Spatial Informatics, China University of Mining and Technology, Xuzhou 221116, China

* Corresponding author, E-mail: yhy@zjnu.cn

Contents

1. **1.** Details of Homology Modeling
2. **2.** Molecular Mechanics/Generalized Born Surface Area (MM/GBSA) methods to calculate binding energy (ΔG_{bind}) between ligands and rAhR
3. **3.** QSAR models study

Fig. S1. Ramachandran plot image of rAhR homology structure based on 3F1O which has the native ligand.

Fig. S2. The ProSA analyses of the generated rAhR LBD structure model.

Fig. S3. Structural aligning the structures of the homology rAhR model (pink loop) and five template models (chain A of 3F1P, 3F1O, 3F1N, 3H7W and chain B of 3H82) on their C*α* atoms.

Fig. S4. The root-mean-square deviation (RMSD, Å) of 15 types of PACs and rAhR.

Fig. S5. The root-mean-square fluctuations (RMSF, Å) of 15 types rAhR bound with PACs.

Fig. S6. The hydrogen bond and *π*-*π* interaction diagram of complexes which 7H-benz[de]anthracen-7-one, 4-nitropyrene and 9,10-dichlorophenanthrene were bound with rAhR.

Fig. S7. The model of rat aryl hydrocarbon receptor (rAhR), the dark frame is binding site.

Table S1. Autodock vina scores of 83 PACs with rAhR.

Table S2. The amino acid residues with energy contributions of 15 types of PACs.

Table S3. The experimental and predictive log *IEQ* values and selected molecular descriptors values in model (1).

Table S4. The experimental and predictive log *%*-TCDD-*max* values and selected molecular descriptors values in model (2).

1. Details of Homology Modeling

We construct the LBD of rAhR through the homology modeling. The amino acid sequence (P41738 [1]: 282-388) of the PAS B domain of rAhR was modeled based on the NMR structures of the human hypoxia-inducible factor 2α (HIF-2α) [2,3]. The sequence of HIF-2α shows the high homology with that of rAhR PAS B domain. Homology models were constructed using Modeller 10.1 [4], based on the template models 3F1P, 3F1O, 3F1N, 3H7W and 3H82 [5,6] obtained from the protein data bank (PDB) database (http://www.rcsb.org/). The 3D model was subjected to energy minimization using UCSF chimera 1.11rc [7].

2. Molecular Mechanics/Generalized Born Surface Area (MM/GBSA) methods to calculate binding energy (ΔG_{bind}) between ligands and rAhR, formulas are as follows [8]:

$$\Delta G_{\mathrm{bind}} = G_{\mathrm{conplex}} - (G_{\mathrm{ligands}} + G_{\mathrm{rAhR}})$$

Equation consists of the following formula:

$$G = E_{\mathrm{MM}} + G_{\mathrm{solv}} - TS$$

$$E_{\mathrm{MM}} = E_{\mathrm{ele}} + E_{\mathrm{vdw}}$$

$$G_{\mathrm{solv}} = G_{\mathrm{GB}} + G_{\mathrm{SA}}$$

Where G_{complex}, G_{ligands} and G_{rAhR} are the binding energy of complex, ligands and rAhR. E_{MM} is the average gas-phase molecular mechanics energy, E_{ele} is the energy of electrostatic, E_{vdw} is the energy of van der Waals interaction, G_{solv} is the electrostatic solvation free energy, G_{GB} and G_{SA} are the free energy of polar and nonpolar solvation, respectively, TS is the entropy contribution, temperature is 300 K.

3. QSAR models study

The geometries of all PACs were optimized by M06-2X functional [9] and Def2-SVP basis set, which was carried out by Gaussian 09 program [10], to obtain the molecular parameters that reflect the molecular energy, charge and surface potential information. The dragon descriptors were calculated by Dragon 6[11]. The octanol-water partitioning coefficient (log K_{OW}) was further employed as a molecular descriptor, which was computed by EPI^{TM} Suite 4.11 [12]. Total 4908 descriptors for each molecule were obtained. Defined log *IEQ* and log %-TCDD-*max* datasets as endpoints, developed two QSAR models were developed by multiple linear regression (MLR) analysis via IBM SPSS Statistics 21 [13] for 62 PACs without halogen and 28 PACs with halogen, respectively. The statistical parameters of the fitting correlation coefficient square (R^2), prediction correlation coefficients square (Q^2), root mean square error (*RMSE*), mean absolute error (*MAE*), maximum positive prediction error (*MPE*), maximum negative prediction error (*MNE*) and systematic error (*BIAS*) [14], were calculated to quantify the statistical performance of the developed models.

Simulated external validation was implemented to assess the predictive ability of developed models as described in the reference [15], in which 70% compounds were randomly selected to destruct new models and were tested by the rest data. Except for R^2 and Q^2 values of training set and test set, statistical parameters Q^2_{F1}, Q^2_{F2}, Q^2_{F3} and concordance correlation coefficient (*CCC*) for validation were also used to evaluate the predictive ability of the models [16,17]. Leave-one-out cross validation [18] was carried out by Weka 3.6 [19] to represent the robustness of the models through the cross validation prediction correlation coefficients (Q^2_{CV}) and cross validation root mean square error ($RMSE_{CV}$). The Q_{F1}^2、Q_{F2}^2、Q_{F3}^2、*CCC* formulas are as follows [16]:

$$Q_{F1}^2 = 1 - \frac{\sum_{i=1}^{n_{TE}} (y_i^{exp} - y_i^{pred})^2}{\sum_{i=1}^{n_{TE}} (y_i^{exp} - \bar{y}_{TR}^{exp})^2}$$

$$Q_{F2}^2 = 1 - \frac{\sum_{i=1}^{n_{TE}} (y_i^{exp} - y_i^{pred})^2}{\sum_{i=1}^{n_{TE}} (y_i^{exp} - \bar{y}_{TE}^{exp})^2}$$

$$Q_{F3}^2 = 1 - \frac{\sum_{i=1}^{n_{TE}} (y_i^{exp} - y_i^{pred})^2 / n_{TE}}{\sum_{i=1}^{\mathrm{n_{TR}}} (y_i^{exp} - \bar{y}_{TR}^{exp})^2 / n_{TR}}$$

$$CCC = \frac{2 \sum_{i=1}^{n_{TE}} (y_i^{exp} - \bar{y}^{exp})(y_i^{pred} - \bar{y}^{pred})}{\sum_{i=1}^{n_{TE}} (y_i^{exp} - \bar{y}^{exp})^2 + \sum_{i=1}^{n_{TE}} (y_i^{pred} - \bar{y}^{pred})^2 + n_{TE}(\bar{y}^{exp} - \bar{y}^{pred})^2}$$

Where y_i^{exp} is the experimental values, $\bar{y}^{exp}$ is the average of experimental values, y_i^{pred} is the predictive values, $\bar{y}^{pred}$ is the average of predictive values, *TR* is the training set and *TE* is test set.

Williams plot was drawn by the leverage value (h_i, x-axis of the 2D graph) and the standardized residual (*SR*, y-axis), to define the application domain and determine outliers. Herein, h_i is calculated by the formula $h_i = x_{\mathrm{i}}^{\mathrm{T}}(X^{\mathrm{T}}X)^{-1}x_i$, in which X is the 2D descriptor matrix comprised of n lines (compounds) and p columns (descriptors) and x_i is the descriptor line vector of the compounds. The compound with $|SR| > 3$ is defined as an outlier, and it should be removed from the model. The higher h_i than h^* ($3p/n$, p and n are the number of descriptors and compounds, respectively) indicates that the corresponding compound may have a large impact on the model regression [20].

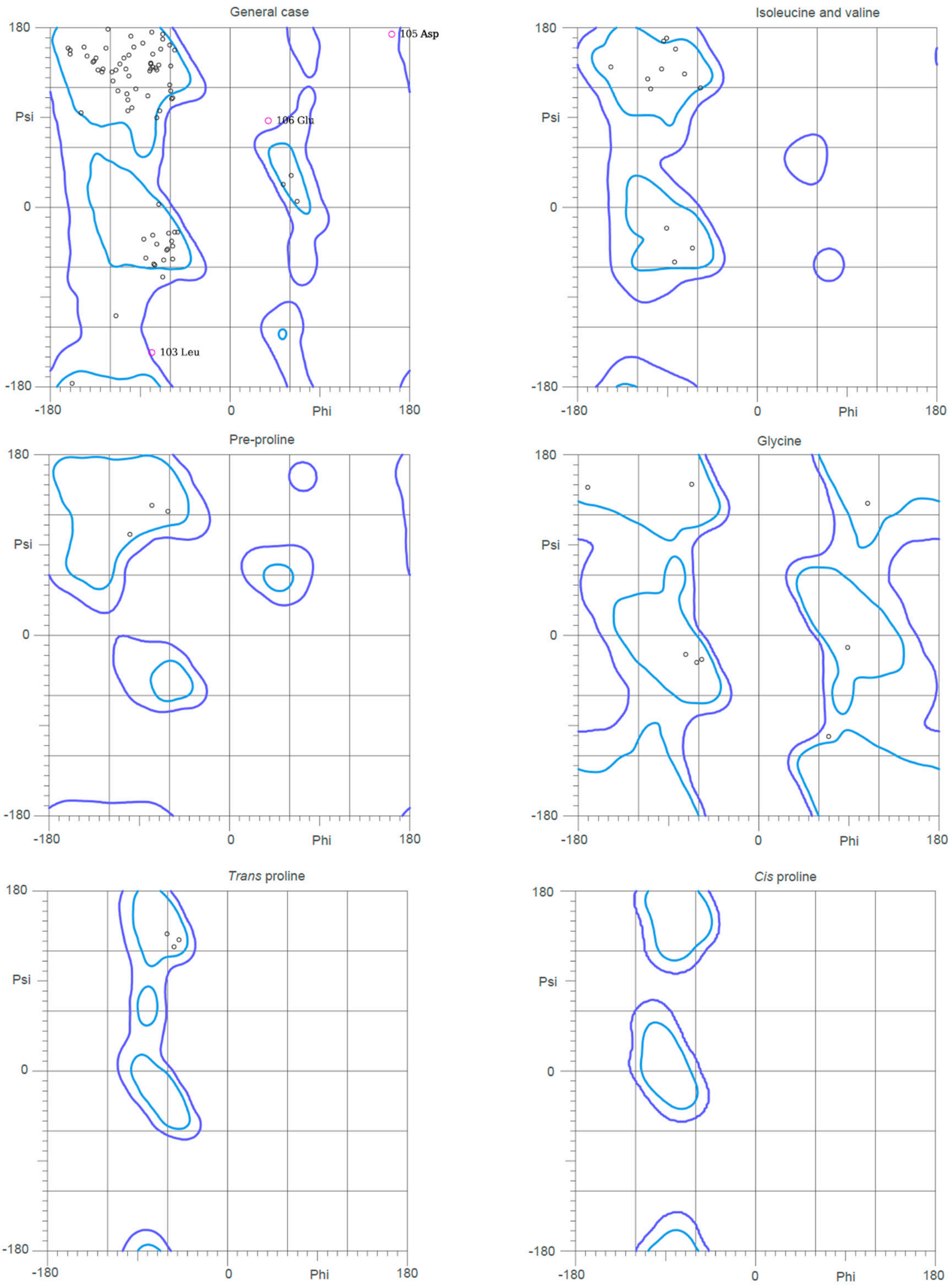

Fig. S1. Ramachandran plot image of rAhR homology structure based on 3F1O which has the native ligand.

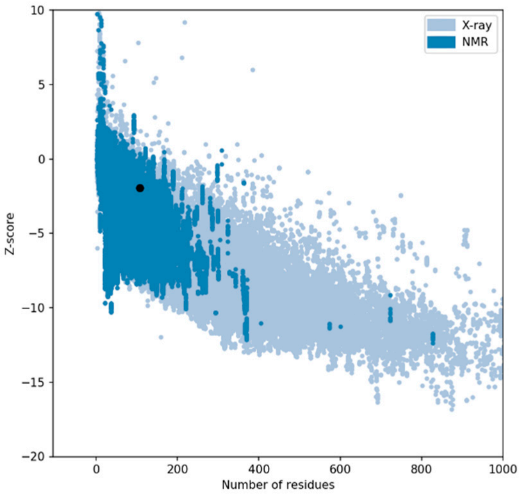

Fig. S2. The ProSA analyses of the generated rAhR LBD structure model. The calculated quality (Z) scores (closed circles) are displayed in the context of all experimentally determined protein structures available in the Protein Data Bank with each dot representing a distinct structure solved by X-ray crystallography (light blue) or NMR (dark blue). The figure represents the prosa-web plot of homology rAhR model with a z-score value of −1.94.

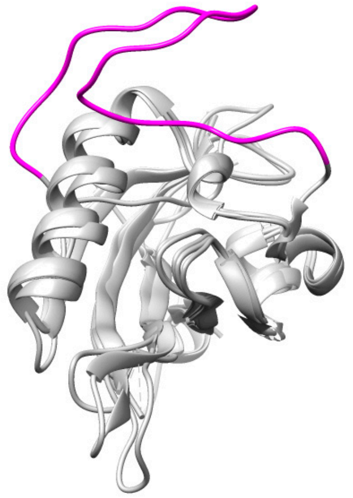

Fig. S3. Structural aligning the structures of the homology rAhR model (pink loop) and five template models (chain A of 3F1P, 3F1O, 3F1N, 3H7W and chain B of 3H82) on their Cα atoms.

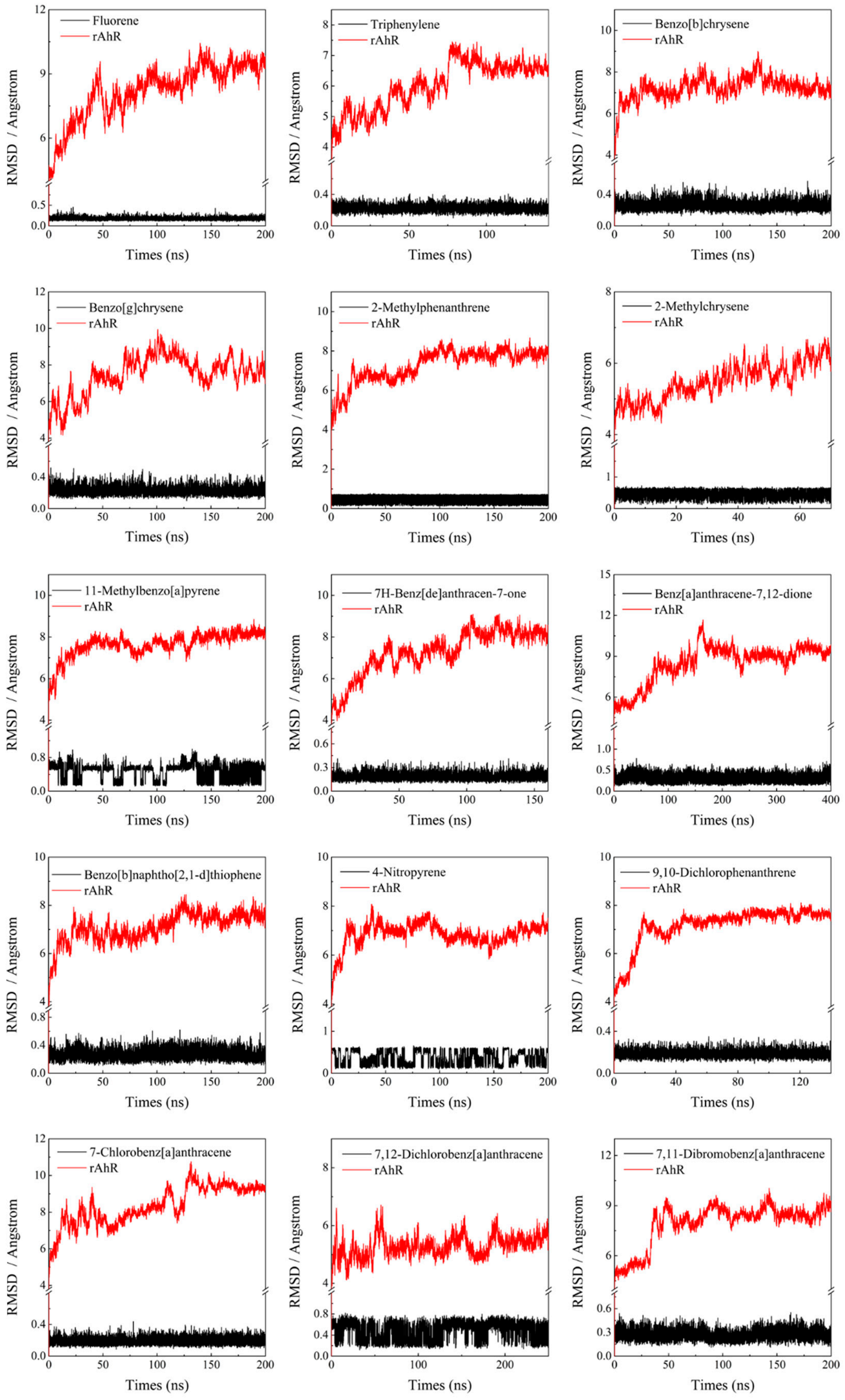

Fig. S4. The root-mean-square deviation (RMSD, Å) of 15 types of PACs and rAhR.

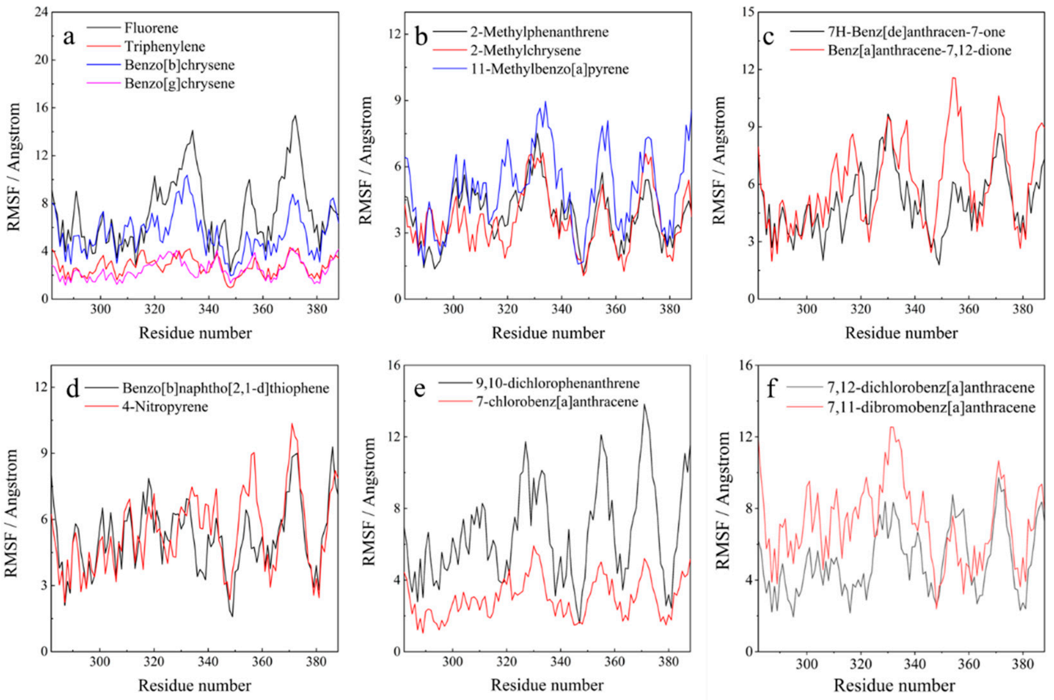

Fig. S5. The root-mean-square fluctuations (RMSF, Å) of 15 types rAhR bound with PACs.

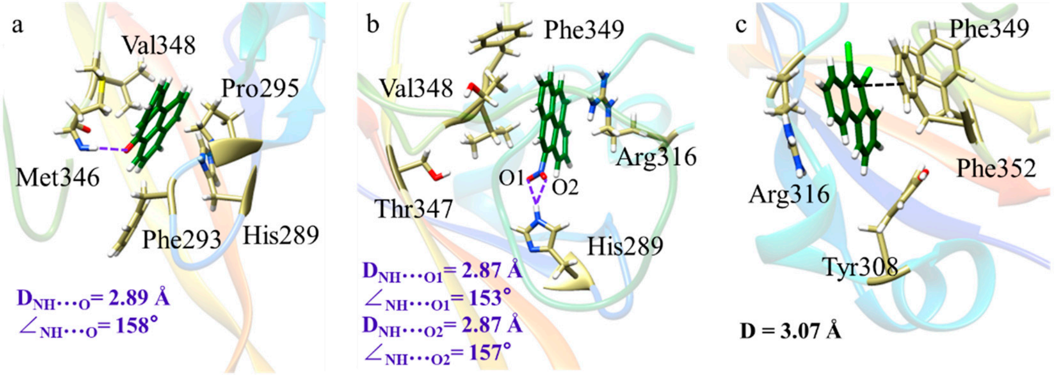

7H-benz[de]anthracen-7-one 4-nitropyrene 9,10-dichlorophenanthrene

Fig. S6. The hydrogen bond (purple dotted line) and *π*-*π* interaction (dark dotted line) diagram of complexes which 7H-benz[de]anthracen-7-one, 4-nitropyrene and 9,10-dichlorophenanthrene were bound with rAhR.

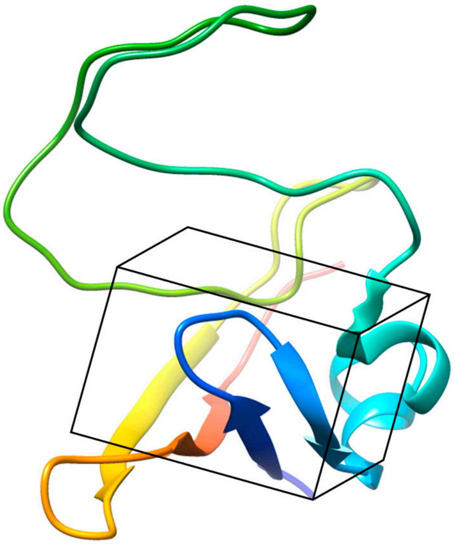

Fig. S7. The model of rat aryl hydrocarbon receptor (rAhR), the dark frame is binding site (angstrom = 1, center_x = 16.525, center_y = -43.151, center_z = 11.693, size_x = 20, size_y = 14, size_z = 20).

Table S1. Autodock vina scores of 83 PACs with rAhR.

No	Coumpounds	CAS	Scores
	PAHs		
1	Fluorene	86-73-7	−7.2
2	Chrysene	218-01-9	−8.5
3	Pyrene	129-00-0	−7.7
4	Benz[a]anthracene	56-55-3	−8.8
5	11H-Benzo[a]fluorene	238-84-6	−8.8
6	11H-Benzo[b]fluorene	243-17-4	−8.5
7	Benzo[c]phenanthrene	195-19-7	−7.1
8	Triphenylene	217-59-4	−6.3
9	Benzo[b]chrysene	214-17-5	−9.6
10	Benzo[c]chrysene	194-69-4	−9.0
11	Benzo[g]chrysene	196-78-1	−7.6
12	Benzo[a]fluoranthene	203-33-8	−7.0
13	Benzo[b]fluoranthene	205-99-2	−7.7
14	Benzo[j]fluoranthene	205-82-3	−8.3
15	Benzo[k]fluoranthene	207-08-9	−8.7
16	Benzo[a]pyrene	50-32-8	−8.7
17	Benzo[e]pyrene	192-97-2	−7.0
18	Dibenz[a,c]anthracene	215-58-7	−7.0
19	Dibenz[a,h]anthracene	53-70-3	−8.2
20	Dibenz[a,j]anthracene	224-41-9	−7.6
21	Picene	213-46-7	−8.3
22	Indeno[1,2,3-cd]pyrene	193-39-5	−7.7
23	Naphtho[2,1-a]pyrene	189-96-8	−7.4
24	Naphtho[1,2-b]fluoranthene	111189-32-3	−7.9
25	Naphtho[1,2-k]fluoranthene	238-04-0	−8.1
26	Naphtho[2,3-b]fluoranthene	206-06-4	−8.1
27	Dibenzo[a,e]fluoranthene	5385-75-1	−7.4
28	Dibenzo[a,k]fluoranthene	84030-79-5	−7.3
29	Dibenzo[b,k]fluoranthene	205-97-0	−7.7
30	Dibenzo[a,l]pyrene	191-30-0	−7.9
31	Dibenzo[a,e]pyrene	192-65-4	−7.8

No	Coumpounds	CAS	Scores
32	Dibenzo[a,i]pyrene	189-55-9	−9.0
33	Dibenzo[a,h]pyrene	189-64-0	−9.5
34	Naphtho[2,3-a]pyrene	196-42-9	−7.2
35	Naphtho[2,3-e]pyrene	193-09-9	−7.5
	M-PAHs		
36	1-Methylanthracene	610-48-0	−8.1
37	2-Methylanthracene	613-12-7	−8.1
38	9-Methylanthracene	779-02-2	−7.8
39	1-Methylphenanthrene	832-69-9	−8.1
40	2-Methylphenanthrene	2531-84-2	−8.0
41	3-Methylphenanthrene	832-71-3	−7.9
42	1-Methylchrysene	3351-28-8	−8.5
43	2-Methylchrysene	3351-32-4	−9.1
44	3-Methylchrysene	3351-31-3	−8.9
45	4-Methylchrysene	3351-30-2	−8.5
46	6-Methylchrysene	1705-85-7	−8.4
47	1-Methylpyrene	2381-21-7	−7.8
48	2-Methylbenz[a]anthracene	2498-76-2	−8.2
49	3-Methylbenz[a]anthracene	2498-75-1	−8.7
50	6-Methybenz[a]anthracene	316-14-3	−8.4
51	7-Methylbenz[a]anthracene	2541-69-7	−8.1
52	10-Methylbenz[a]anthracene	2381-15-9	−8.0
53	6-Methylbenzo[a]pyrene	2381-39-7	−7.6
54	10-Methylbenzo[a]pyrene	63104-32-5	−7.9
55	11-Methylbenzo[a]pyrene	16757-80-5	−7.5
	O-PAHs		
56	7H-Benz[de]anthracen-7-one	82-05-3	−7.1
57	Benz[a]anthracene-7,12-dione	2498-66-0	−7.6
	S-PAHs		
58	Benzo[b]naphtho[1,2-d]thiophene	205-43-6	−7.1
59	Benzo[b]naphtho[2,1-d]thiophene	239-35-0	−7.8
60	Benzo[b]naphtho[2,3-d]thiophene	243-46-9	−7.4
	N-PAHs		

No	Coumpounds	CAS	Scores
61	1-Nitropyrene	5522-43-0	−7.5
62	4-Nitropyrene	57835-92-4	−7.2
	Cl-PAHs		
63	9-Monochlorofluorene	6630-65-5	−7.3
64	2-Monochloroanthracene	17135-78-3	−7.8
65	9-Monochloroanthracene	716-53-0	−7.4
66	1,9-Dichlorophenanthrene	1006693-48-6	−7.6
67	3,9-Dichlorophenanthrene	7473-66-7	−7.8
68	9,10-Dichlorophenanthrene	17219-94-2	−6.1
69	3,9,10-Trichlorophenanthrene	800409-57-8	−6.1
70	3,4-Dichlorofluoranthene	108079-33-0	−7.6
71	3,8-Dichlorofluoranthene	25911-52-8	−7.8
72	1-Monochloropyrene	34244-14-9	−7.7
73	6-Chlorochrysene	95791-46-1	−7.9
74	6,12-Dichlorochrysene	144757-71-1	−7.0
75	7-Chlorobenz[a]anthracene	20268-52-4	−8.0
76	7,12-Dichlorobenz[a]anthracene	63021-10-3	−7.3
77	6-Monochlorobenzo[a]pyrene	21248-01-1	−6.6
	Br-PAHs		
78	7-Monobromobenz[a]anthracene	32795-84-9	−7.3
79	4,7-Dibromobenz[a]anthracene	94210-35-2	−6.0
80	5,7-Dibromobenz[a]anthracene	1006693-52-2	−4.9
81	7,11-Dibromobenz[a]anthracene	1006693-50-0	−5.5
82	7,12-Dibromobenz[a]anthracene	152678-24-5	−6.3
83	6-Monobromobenzo[a]pyrene	21248-00-0	−6.8

Table S2. The amino acid residues with energy (kcal/mol) contributions of 15 types of PACs.

Compounds	Types	Binding energy	Benzene ring	E_{vdw}	E_{ele}	G_{SA}	G_{GB}	TS	Key residues (contribution energy: kcal/mol)
Fluorene	PAHs	−15.24	2	−27.42	−1.22	−3.67	2.80	−14.43	Arg350(−3.05), Leu302(−1.43), Ile301(−1.26), Phe285(−1.11), Leu351(−1.10)
Triphenylene	PAHs	−19.35	3	−32.67	−1.35	−4.46	3.48	−15.77	Ile323(−2.41), Pro295(−2.21), Thr294(−1.89), Phe349(−1.14)
Benzo[b]chrysene	PAHs	−24.02	5	−39.38	−0.78	−5.04	3.76	−17.58	Arg316(−2.68), Phe349(−2.23), Phe322(−1.95), Phe352(−1.93), Leu313(−1.48), Gly317(−1.31), Ser318(−1.28), Thr294(−1.14), Pro295(−1.11)
Benzo[g]chrysene	PAHs	−26.39	5	−39.92	−1.38	−5.27	3.83	−16.57	Tyr308(−2.60), Ile339(−2.28), Val348(−2.11), Pro295(−1.83)
2-methylphenanthrene	M-PAHs	−7.37	3	−22.03	−1.83	−3.53	3.46	−16.63	Arg316(−2.72), Leu313(−1.37), Leu302(−1.36), Arg350(−1.02)
2-methylchrysene	M-PAHs	−19.10	4	−34.42	−0.45	−4.88	2.85	−17.93	His289(−1.87), Val348(−1.69), Pro295(−1.55), Phe293(−1.54), Met346(−1.11), Leu313(−1.09)
11-methylbenzo[a]pyrene	M-PAHs	−17.46	5	−30.64	−3.03	−4.25	4.81	−15.82	Arg316(−4.81), Arg350(−4.13), Met346(−1.08)
7H-benz[de]anthracen-7-one	O-PAHs	−19.91	4	−32.89	−1.8	−4.39	3.72	−15.59	Met346(−2.7), Pro295(−1.90), Phe293(−1.47), Val348(−1.40), His289(−1.38)

Compounds	Types	Binding energy	Benzene ring	E_{vdw}	E_{ele}	G_{SA}	G_{GB}	TS	Key residues (contribution energy: kcal/mol)
7,12-benzo[a]anthraquinone	O-PAHs	−21.06	4	−36.95	−1.21	−4.45	3.69	−18.02	Tyr308(−4.37), Pro295(−1.83), Tyr320(−1.59), Thr347(−1.46), Leu313(−1.23), Met346(−1.10)
Benzo[b]naphtho[2,1-d]thiophene	S-PAHs	−18.37	3	−32.67	−1.65	−4.13	3.62	−16.64	Phe349(−2.54), Pro295(−1.97), Thr347(−1.70), Thr294(−1.58), Val348(−1.15)
4-nitropyrene	N-PAHs	−18.11	4	−32.30	−3.16	−4.49	4.99	−17.19	Arg316(−2.52), Val348(−2.51), Phe349(−1.74), Thr347(−1.22)
9,10-dichlorophenanthrene	Cl-PAHs	−13.56	3	−25.87	−1.16	−3.62	2.67	−14.52	Arg316(−3.60), Phe352(−3.59), Tyr308(−1.27), Phe349(−1.18)
7-chlorobenz[a]anthracene	Cl-PAHs	−22.84	4	−36.33	−1.00	−4.69	3.22	−16.08	Phe349(−2.97), Leu313(−1.56), Pro295(−1.39), Val348(−1.39), Phe352(−1.30), Tyr308(−1.23), Arg350(−1.21), Leu351(−1.17), Leu302(−1.02)
7,12-dichlorobenz[a]anthracene	Cl-PAHs	−25.06	4	−38.79	−1.65	−4.90	3.72	−16.71	Arg316(−2.89), Phe349(−2.79), Tyr308(−1.81), Pro295(−1.79), Val348(−1.78), Leu313(−1.45)
7,11-dibromobenz[a]anthracene	Br-PAHs	−26.28	4	−38.73	−1.84	−4.82	3.94	−15.30	Arg316(−4.90), Phe349(−3.36), Arg350(−1.83), Pro295(−1.25)

Table S3. The experimental and predictive log *IEQ* values and selected molecular descriptors values in model (1).

No	Compounds	Log *IEQ* Exp.	Log *IEQ* Pred.	$SpMin_{2Bh(m)}$	$HATS_{5p}$	σ	$MATS_{5s}$	H_{6e}	E_{2v}	$SpMax_{8Bh(i)}$
1	Benzo[k]fluoranthene[a]	3.62	3.57	2.14	0.09	9.73	−0.03	0.24	0.26	2.67
2	Indeno[1,2,3-cd]pyrene	3.29	2.52	2.06	0.11	10.52	−0.09	0.20	0.28	2.72
3	Chrysene	3.13	2.48	2.01	0.11	8.70	−0.13	0.33	0.25	2.49
4	Benzo[b]chrysene	3.12	2.06	2.10	0.09	10.07	−0.03	0.49	0.25	2.77
5	Benzo[j]fluoranthene[a]	3.07	3.08	2.14	0.11	10.05	−0.11	0.31	0.28	2.72
6	Naphtho[1,2-k]fluoranthene[a]	2.99	2.50	2.14	0.25	9.82	−0.09	0.24	0.28	2.78
7	3-Methylchrysene[a]	2.97	1.83	2.02	0.12	8.71	−0.03	0.34	0.27	2.65
8	Benzo[c]chrysene[a]	2.95	2.36	2.09	0.22	8.74	−0.08	0.25	0.28	2.72
9	Naphtho[2,3-b]fluoranthene[a]	2.90	2.07	2.06	0.08	10.07	−0.03	0.47	0.25	2.85
10	Dibenz[a,h]anthracene	2.74	2.85	2.10	0.08	9.30	−0.03	0.41	0.26	2.79
11	Dibenzo[b,k]fluoranthene[a]	2.70	3.78	2.17	0.08	9.70	0.01	0.37	0.25	2.91
12	3-Methylbenz[a]anthracene[a]	2.59	2.18	2.03	0.12	9.55	0.00	0.26	0.25	2.67

No	Compounds	Log *IEQ* Exp.	Log *IEQ* Pred.	$SpMin_{2Bh(m)}$	$HATS_{5p}$	σ	$MATS_{5s}$	H_{6e}	E_{2v}	$SpMax_{8Bh(i)}$
13	6-Methylchrysene[a]	2.42	2.46	2.02	0.16	8.81	−0.15	0.36	0.26	2.74
14	Dibenz[a,c]anthracene[a]	2.42	2.26	2.05	0.08	9.27	−0.01	0.32	0.29	2.88
15	Naphtho[2,1-a]pyrene[a]	2.41	2.31	2.12	0.10	10.45	−0.09	0.46	0.27	2.81
16	7-Methylbenz[a]anthracene[a]	2.37	1.44	2.02	0.19	9.71	−0.14	0.34	0.27	2.70
17	Benzo[b]fluoranthene[a]	2.36	2.82	2.06	0.11	9.14	−0.09	0.29	0.26	2.55
18	2-Methylchrysene[a]	2.31	2.22	2.02	0.13	8.69	−0.13	0.48	0.25	2.72
19	Naphtho[1,2-b]fluoranthene[a]	2.12	2.42	2.06	0.09	9.76	−0.12	0.40	0.29	2.85
20	Benzo[a]pyrene[a]	2.07	2.08	2.02	0.12	10.46	−0.08	0.22	0.26	2.68
21	Fluorene[a]	0.04	−0.74	1.97	0.26	7.62	0.05	0.13	0.26	1.72
22	Pyrene[a]	1.91	1.46	1.86	0.14	9.46	−0.11	0.00	0.27	2.43
23	Benz[a]anthracene[a]	1.66	1.99	2.02	0.09	9.51	0.02	0.19	0.26	2.47
24	11H-Benzo[a]fluorene[a]	0.94	0.39	2.00	0.27	8.57	−0.01	0.25	0.25	2.40
25	11H-Benzo[b]fluorene[a]	0.28	0.74	2.00	0.19	8.61	0.04	0.25	0.25	2.37

No	Compounds	Log *IEQ* Exp.	Log *IEQ* Pred.	$SpMin_{2Bh(m)}$	$HATS_{5p}$	σ	$MATS_{5s}$	H_{6e}	E_{2v}	$SpMax_{8Bh(i)}$
26	Benzo[c]phenanthrene	0.26	0.69	2.00	0.25	8.77	0.00	0.07	0.30	2.48
27	Triphenylene[a]	0.11	0.02	1.88	0.13	7.81	−0.09	0.26	0.32	2.44
28	Benzo[a]fluoranthene[a]	0.63	0.80	2.07	0.11	10.97	0.03	0.24	0.30	2.62
29	Benzo[e]pyrene	0.58	0.93	1.94	0.13	9.16	−0.03	0.22	0.29	2.61
30	Benzo[g]chrysene[a]	0.93	1.36	2.03	0.22	8.91	−0.03	0.25	0.29	2.82
31	Dibenz[a,j]anthracene	1.77	1.20	2.03	0.22	9.23	−0.03	0.25	0.29	2.82
32	Picene[a]	1.48	2.59	2.10	0.09	8.72	−0.14	0.61	0.26	2.77
33	Dibenzo[a,e]fluoranthene[a]	0.11	0.49	2.10	0.19	10.59	0.04	0.31	0.31	2.88
34	Dibenzo[a,k]fluoranthene[a]	2.50	2.42	2.17	0.07	11.36	0.08	0.26	0.28	2.86
35	Dibenzo[a,l]pyrene[a]	0.00	0.10	2.03	0.22	10.35	0.05	0.25	0.29	2.82
36	Dibenzo[a,e]pyrene	1.32	1.17	2.04	0.10	10.05	−0.04	0.38	0.31	2.91
37	Dibenzo[a,i]pyrene[a]	1.67	1.30	2.09	0.10	10.76	−0.06	0.56	0.26	2.83
38	Dibenzo[a,h]pyrene[a]	0.72	0.46	2.09	0.11	11.73	−0.06	0.64	0.26	2.87

No	Compounds	Log *IEQ* Exp.	Log *IEQ* Pred.	$SpMin_{2Bh(m)}$	$HATS_{5p}$	σ	$MATS_{5s}$	H_{6e}	E_{2v}	$SpMax_{8Bh(i)}$
39	Naphtho[2,3-a]pyrene	1.15	1.80	2.12	0.09	11.74	−0.03	0.40	0.26	2.81
40	Naphtho[2,3-e]pyrene	1.83	2.72	2.09	0.09	9.25	0.04	0.32	0.27	2.87
41	1-Methylanthracene[a]	0.08	0.04	1.93	0.17	9.86	0.01	0.12	0.27	2.35
42	2-Methylanthracene	0.20	0.17	1.93	0.12	9.80	0.07	0.21	0.25	2.37
43	9-Methylanthracene[a]	−1.74	−0.19	1.90	0.27	10.03	−0.20	0.22	0.25	2.26
44	1-Methylphenanthrene	0.72	0.75	1.90	0.20	8.13	−0.15	0.22	0.28	2.37
45	2-Methylphenanthrene[a]	1.43	2.00	1.91	0.15	7.99	−0.11	0.19	0.25	2.38
46	3-Methylphenanthrene[a]	0.77	1.05	1.91	0.13	8.05	0.01	0.09	0.28	2.37
47	1-Methylpyrene	0.65	0.42	1.89	0.19	9.65	−0.08	0.08	0.29	2.53
48	6-Methybenz[a]anthracene[a]	2.73	2.27	2.02	0.13	9.53	−0.03	0.26	0.24	2.62
49	2-Methylbenz[a]anthracene[a]	1.58	1.69	2.03	0.10	9.59	0.10	0.16	0.27	2.61
50	10-Methylbenz[a]anthracene	1.34	1.58	2.03	0.10	9.50	0.01	0.30	0.27	2.66
51	1-Methylchrysene[a]	1.25	1.26	2.02	0.16	8.77	−0.15	0.46	0.28	2.61

No	Compounds	Log *IEQ* Exp.	Log *IEQ* Pred.	$SpMin_{2Bh(m)}$	$HATS_{5p}$	σ	$MATS_{5s}$	H_{6e}	E_{2v}	$SpMax_{8Bh(i)}$
52	4-Methylchrysene[a]	1.68	1.76	2.02	0.17	8.91	−0.23	0.48	0.27	2.63
53	6-Methylbenzo[a]pyrene[a]	0.11	0.44	2.02	0.21	10.60	−0.15	0.40	0.28	2.73
54	10-Methylbenzo[a]pyrene	1.53	1.06	2.03	0.17	10.58	−0.21	0.40	0.29	2.69
55	11-Methylbenzo[a]pyrene[a]	−0.40	0.22	2.02	0.17	10.58	−0.09	0.47	0.27	2.72
56	7H-Benz[de]anthracen-7-one[a]	0.75	0.77	1.93	0.13	9.71	−0.08	0.16	0.30	2.59
57	Benz[a]anthracene-7,12-dione	−0.70	−0.35	1.99	0.28	9.85	0.02	0.11	0.29	2.55
58	Benzo[b]naphtho[1,2-d]thiophene[a]	0.28	0.38	1.96	0.13	8.69	−0.02	0.19	0.30	2.27
59	Benzo[b]naphtho[2,1-d]thiophene	1.22	1.90	1.98	0.11	8.57	−0.07	0.19	0.27	2.28
60	Benzo[b]naphtho[2,3-d]thiophene	1.05	0.66	1.98	0.11	9.21	0.02	0.18	0.29	2.35
61	1-Nitropyrene[a]	0.89	0.48	1.86	0.13	10.25	−0.11	0.10	0.29	2.53
62	4-Nitropyrene	−1.40	−1.39	1.86	0.13	10.14	−0.04	0.13	0.34	2.50

Note: [a] forms the training set, Log *IEQ* Exp. are the experimental values of log *IEQ* and Log *IEQ* Pred. are the predictive values of log *IEQ*.

Table S4. The experimental and predictive log %-TCDD-*max* values and selected molecular descriptors values in model (2).

No	Compounds	Log %-TCDD-*max* experimental values	Log %-TCDD-*max* predictive values	*HGM*	E_{HOMO}	$ATSC_{1e}$
1	9-Monochlorofluorene[a]	−0.05	0.01	7.86	−0.28	0.01
2	2-Monochloroanthracene	0.11	0.10	6.62	−0.25	0.01
3	9-Monochloroanthracene[a]	−0.05	0.02	6.60	−0.25	0.01
4	1,9-Dichlorophenanthrene	0.70	0.97	6.24	−0.27	0.00
5	3,9-Dichlorophenanthrene[a]	1.40	0.96	6.19	−0.27	0.00
6	9,10-Dichlorophenanthrene[a]	0.93	0.96	6.22	−0.27	0.00
7	3,9,10-Trichlorophenanthrene[a]	1.77	1.82	6.13	−0.27	0.03
8	3,4-Dichlorofluoranthene[a]	1.26	1.25	5.71	−0.27	0.00
9	3,8-Dichlorofluoranthene	1.59	1.36	5.71	−0.27	0.00
10	1-Monochloropyrene	0.79	0.82	5.72	−0.25	0.01
11	6-Chlorochrysene	1.90	1.76	5.07	−0.26	0.01
12	6,12-Dichlorochrysene[a]	1.23	1.54	5.01	−0.26	0.00
13	7-Chlorobenz[a]anthracene[a]	1.85	1.71	4.80	−0.25	0.01
14	7,12-Dichlorobenz[a]anthracene[a]	1.15	0.80	5.60	−0.25	0.00
15	6-Monochlorobenzo[a]pyrene[a]	1.40	1.51	4.62	−0.24	0.01
16	7-Monobromobenz[a]anthracene[a]	1.92	1.81	4.79	−0.25	0.02

No	Compounds	Log %-TCDD-*max* experimental values	Log %-TCDD-*max* predictive values	*HGM*	E_{HOMO}	$ATSC_{1e}$
17	4,7-Dibromobenz[a]anthracene[a]	1.97	1.80	4.75	−0.26	0.01
18	5,7-Dibromobenz[a]anthracene[a]	1.65	1.79	4.73	−0.26	0.01
19	7,11-Dibromobenz[a]anthracene[a]	1.43	1.80	4.73	−0.26	0.01
20	7,12-Dibromobenz[a]anthracene[a]	0.75	1.00	5.65	−0.26	0.01
21	6-Monobromobenzo[a]pyrene	1.78	1.61	4.61	−0.24	0.02

Note: [a] forms the training set.

Reference

1. Medicine, N L O. UniProtKB/Swiss-Prot. United States government, 2003.UniProtKB/Swiss-Prot. Available online: https://www.expasy.org/resources/uniprotkb-swiss-prot. (accessed on 10 September 2024).
2. Motto, I.; Bordogna, A.; Soshilov, A A.; Denison, M S.; Bonati, L. New aryl hydrocarbon receptor homology model targeted to improve docking reliability. *J. Chem. Inf. Model*. **2011**, *51*(11), 2868-81.https://doi.org/10.1021/ci2001617.
3. Erbel, P J.; Card, P B.; Karakuzu, O.; Bruick, R K.; Gardner, K H. Structural basis for PAS domain heterodimerization in the basic helix-loop-helix-PAS transcription factor hypoxia-inducible factor. *Proc. Natl. Acad. Sci. U. S. A*. **2003**, *100*(26), 15504-9.https://doi.org/10.1073/pnas.2533374100.
4. Andra´ S Fiser, A S A. Modeller_ Generation and Refinement of Homology-Based Protein Structure Models. *Methods in Enzymology*. **2003**, *374*, 461-491
5. Nuti, R.; Gargaro, M.; Matino, D.; Dolciami, D.; Grohmann, U.; Puccetti, P.; Fallarino, F.; Macchiarulo, A. Ligand binding and functional selectivity of L-tryptophan metabolites at the mouse aryl hydrocarbon receptor (mAhR). *J Chem Inf Model*. **2014**, *54*(12), 3373-83.https://doi.org/10.1021/ci5005459.
6. Faber, S C.; Giani Tagliabue, S.; Bonati, L.; Denison, M S. The Cellular and Molecular Determinants of Naphthoquinone-Dependent Activation of the Aryl Hydrocarbon Receptor. *Int J Mol Sci*. **2020**, *21*(11).https://doi.org/10.3390/ijms21114111.
7. Mohaddeseh, B.; Ibrahim, T.; Mehrdad, M.; Gholamreza, A.; Andrew J, E. Comparative modeling of CCRL1, a key protein in masked immune diseases and virtual screening for finding inhibitor of this protein. *Bioinformation*. **2012**, *8*(7), 336-340.https://doi.org/10.6026/97320630008336.
8. Xu, L.; Sun, H.; Li, Y.; Wang, J.; Hou, T. Assessing the performance of MM/PBSA and MM/GBSA methods. 3. The impact of force fields and ligand charge models. *J Phys Chem B*. **2013**, *117*(28), 8408-21.https://doi.org/10.1021/jp404160y.
9. Yan Zhao, D G T. The M06 suite of density functionals for main group thermochemistry, thermochemical kinetics, noncovalent interactions, excited states, and transition elements two new functionals. *Theoretical Chemistry Accounts*. **2008**, *120*, 215-241.https://doi.org/10.1007/s00214-007-0310-x.
10. Frisch, M J, Trucks, G.W., Schlegel, H.B., Scuseria, G.E., Robb, M.A.,.; Cheeseman, J R, Scalmani, G., Barone, V., Mennucci, B.,.; Petersson, G A, Nakatsuji, H., Caricato, M., Li, X., Hratchian,.; H.P., I, A.F., Bloino, J., Zheng, G., Sonnenberg, J.L.,.; Hada, M, Ehara, M., Toyota, K., Fukuda, R., Hasegawa, J.,.; Ishida, M, Nakajima, T., Honda, Y., Kitao, O., Nakai, H.,.; Vreven, T, Montgomery Jr., J.A., Peralta, J.E., Ogliaro, F.,.; Bearpark, M, Heyd, J.J., Brothers, E., Kudin, K.N., Staroverov,.; V.N., K, R., Normand, J., Raghavachari, K., Rendell, A.,.; Burant, J C, Iyengar, S.S., Tomasi, J., Cossi, M. Rega, Millam,.; N.J., K, M., Knox, J.E., Cross, J.B., Bakken, V., Adamo, C.,.; Jaramillo, J, Gomperts, R.E., Stratmann, O., Yazyev, A.J.,.; Austin, R, Cammi, C., Pomelli, J.W., Ochterski, R., Martin, R.L.,.; Morokuma, K, Zakrzewski, V.G., Voth, G.A., Salvador, P.,.; Dannenberg, J J, Dapprich, S., Daniels, A.D., Farkas, O.,.; Foresman, J B, Ortiz, J.V., Cioslowski, J., Fox, D.J.,. Gaussian 09. Revision A. 1. Wallingford, CT, 2009.Gaussian 09. Revision A. 1
11. Talete, S., Dragon for windows (software for molecular descriptor calculations), version 6. http://www.talete.mi.it/products/dragon_description.htm.Dragon for windows (software for molecular descriptor calculations), version 6. (accessed on 10 September 2024).
12. Epa, U. Estimation programs interface suite[TM] for microsoft windows. Washington DC, USA: United States Environmental Protection Agency, 2012.Estimation programs interface suite[TM] for microsoft windows
13. Hashemianzadeh, M.; Safarpour, M A.; Gholamjani-Moghaddam, K.; Mehdipour, A R. DFT-Based QSAR Study of Valproic Acid and its Derivatives. *QSAR & Combinatorial Science*. **2008**, *27*(4), 469-474.https://doi.org/10.1002/qsar.200710093.
14. Development, O F E C-O A. Guidance Document on the Validation of (Quantitative) Structure-Activity Relationship [(Q)SAR] Models. *OECD Environment Health and Safety Publications Series on Testing and Assessment*. **2007**, *69. OECD: Paris, France, 2014.*
15. Wei, X.; Yuan, Q.; Serge, B.; Xu, T.; Ma, G.; Yu, H. In silico investigation of gas/particle partitioning equilibrium of polybrominated diphenyl ethers (PBDEs). *Chemosphere*. **2017**, *188*, 110-118.https://doi.org/10.1016/j.chemosphere.2017.08.146.
16. Chirico, N.; Gramatica, P. Real External Predictivity of QSAR Models. Part 2. New Intercomparable Thresholds for Different Validation Criteria and the Need for Scatter Plot Inspection. *Journal of Chemical Information and Modeling*. **2012**, *52*(8), 2044-2058.https://doi.org/10.1021/ci300084j.

17. Todeschini, R.; Ballabio, D.; Grisoni, F. Beware of unreliable Q2! A comparative study of regression metrics for predictivity assessment of QSAR models. *J. Chem. Inf. Model*. **2016**, *56*(10), 1905-1913.https://doi.org/10.1021/acs.jcim.6b00277.
18. Eriksson, L.; Jaworska, J.; Worth, A P.; Cronin, M T D.; Mcdowell, R M.; Gramatica, P. Methods for reliability and uncertainty assessment and for applicability evaluations of classification- and regression-based QSARs. *Environmental Health Perspectives*. **2003**, *111*(10), 1361-1375.https://doi.org/10.1289/ehp.5758.
19. Mark, H.; Eibe, F.; Geoffrey, H.; Bernhard, P.; Peter, R.; Ian H., W. The Weka data mining software: an update. *SIGKDD Explorations*. **2009**, *11*(1), 10-18.https://doi.org/10.1145/1656274.1656278.
20. Saavedra, L M.; Duchowicz, P R. Predicting zebrafish (Danio rerio) embryo developmental toxicity through a non-conformational QSAR approach. *Science of The Total Environment*. **2021**, *796*, 148820.https://doi.org/10.1016/j.scitotenv.2021.148820.
